# Heterojunction semiconductor nanocatalysts as cancer theranostics

**DOI:** 10.1063/5.0223718

**Published:** 2024-10-04

**Authors:** Arjun Sabu, Manoj Kandel, Ritwick Ranjan Sarma, Lakshminarayan Ramesan, Ekta Roy, Ramalingam Sharmila, Hsin-Cheng Chiu

**Affiliations:** Department of Biomedical Engineering and Environmental Sciences, National Tsing Hua University, Hsinchu, Taiwan

## Abstract

Cancer nanotechnology is a promising area of cross-disciplinary research aiming to develop facile, effective, and noninvasive strategies to improve cancer diagnosis and treatment. Catalytic therapy based on exogenous stimulus-responsive semiconductor nanomaterials has shown its potential to address the challenges under the most global medical needs. Semiconductor nanocatalytic therapy is usually triggered by the catalytic action of hot electrons and holes during local redox reactions within the tumor, which represent the response of nontoxic semiconductor nanocatalysts to pertinent internal or external stimuli. However, careful architecture design of semiconductor nanocatalysts has been the major focus since the catalytic efficiency is often limited by facile hot electron/hole recombination. Addressing these challenges is vital for the progress of cancer catalytic therapy. In recent years, diverse strategies have been developed, with heterojunctions emerging as a prominent and extensively explored method. The efficiency of charge separation under exogenous stimulation can be heightened by manipulating the semiconducting performance of materials through heterojunction structures, thereby enhancing catalytic capabilities. This review summarizes the recent applications of exogenous stimulus-responsive semiconducting nanoheterojunctions for cancer theranostics. The first part of the review outlines the construction of different heterojunction types. The next section summarizes recent designs, properties, and catalytic mechanisms of various semiconductor heterojunctions in tumor therapy. The review concludes by discussing the challenges and providing insights into their prospects within this dynamic and continuously evolving field of research.

## I. INTRODUCTION

Catalysis is a vital process of biochemical reactions, playing a pivotal role in orchestrating numerous metabolic pathways and physiological functions within our bodies with remarkable precision and efficiency.^1,2^ In catalysis, a key player, a catalytic agent or catalyst, accelerates a chemical reaction by lowering the activation energy without being used itself. This efficiency is crucial for various biochemical processes, including metabolism, digestion, energy production, detoxification, DNA maintenance, and cellular signaling.^3^ Catalytic medicine is an exciting new field utilizing this concept for therapeutic purpose. Instead of traditional drugs, it uses nontoxic artificial catalysts, especially metal-containing or metal based nanoparticles (NPs) known as nanozymes, to treat diseases.^4–7^ These catalysts work by accelerating desired reactions at the disease site, producing beneficial molecules that promote healing. Scientists are interested in this new approach to fighting cancer using specially designed entities called “catalytic nanomedicines.” These entities target unique chemical features found within tumors, possibly improving treatment and reducing drug resistance. Our bodies naturally produce powerful oxidizing molecules such as hydroxyl radical (•OH) through hydrogen peroxide (H_2_O_2_) breakdown catalyzed by metal ions. Similarly, peroxidase (POD)-like nanozymes were used to create •OH radicals from endogenously overexpressed H_2_O_2_ in tumor microenvironment (TME) to kill cancer cells.^8^ Based on their architecture and properties, nanozymes show different catalytic activities, such as POD-, catalase (CAT)-, superoxide dismutase (SOD)-, glucose oxidase (GOx)-, and glutathione peroxidase (GPx)-like catalytic activities.^9^ However, this self-sustained activity faces challenges related to targeting and specificity issues, as nanozymes can cause off-target effects and toxicity to other organs, underscoring the importance of precise targeting strategies and thorough biocompatibility assessments for their successful implementation in clinical settings.

Catalytic therapy can be further enhanced by introducing external triggers, such as light, temperature, ultrasound (US), and magnetic force, a strategy called exogenous stimulation therapy. This approach offers several advantages compared to endogenous therapy. Unlike relying solely on the body's natural catalysts, exogenous stimulation therapy utilizes various catalytic substrates, increasing both efficiency and effectiveness. The success of this approach depends on the carefully chosen catalyst and its unique physical and chemical properties. Selecting the right catalyst ensures optimal performance and maximizes the therapeutic potential of exogenous stimulation therapy. The recent surge in research has focused on the synergy between specific semiconductors and external energy sources, such as light, US, temperature, and magnetic force. This powerful combination is promising for generating clean energy sources and decontaminating water. A key discovery by researchers K. Honda and A. Fujishima substantially advanced the photocatalysis field (light as the stimuli) in 1972.^10^ They demonstrated that titanium dioxide (TiO_2_) could split water molecules when exposed to light. This breakthrough laid the foundation for further exploration of photocatalysis. Meanwhile, sonocatalysis and radiocatalysis utilize sound or radio waves, respectively, to activate semiconducting materials as catalysts. When the catalysts are exposed to these external stimuli with energy higher than their band gaps (h*ν* ≥ E_g_), an electron (e^−^) in the catalyst valence band (VB) gets promoted to the conduction band (CB). This creates a hole (h^+^) in the VB, forming an electron–hole pair known as an exciton. These excited electrons and holes are the key players responsible for various chemical reactions. However, a major obstacle in optimizing these catalytic processes is the tendency of excitons to recombine. This recombination, where the excited electron and hole reunite, releases energy and reduces overall efficiency. Therefore, strategies to minimize electron–hole recombination and extend exciton lifetime are crucial for maximizing the performance of these technologies. Following the discovery of interparticle electron transfer by Serpone *et al.* in 1984, pairing semiconductors became a popular strategy in photo-, sono-, and radiocatalysis. This approach suppresses charge recombination by facilitating electron transfer between materials. A decade later, the confirmation of interparticle hole transfer further advanced the research on integrating metal NPs or forming heterojunctions with semiconductors to improve catalytic efficiency.^11^

**SCHEME 1.**
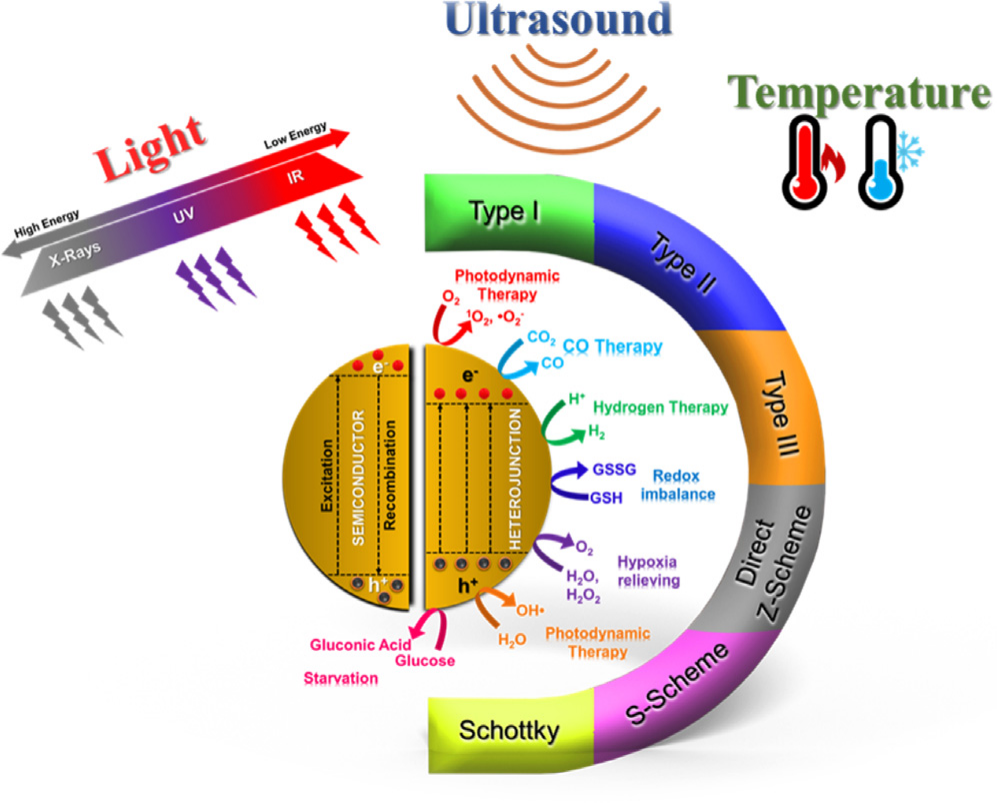
A schematic representation of the classification, stimuli, and modes of cancer treatment using heterojunction nanocatalysts.

Cancer remains a global issue, demanding innovative and well-tolerated treatment.^12^ However, effective, traditional methods, such as surgery, chemotherapy, and radiation therapy, often inflict significant side effects or limitations on patients.^13^ A new therapeutic strategy for cancer treatment based on the *in situ* production of therapeutic compounds or molecules within the tumor, which can effectively kill cancer cells or impair their proliferation, is under development.^14^ Thus, the strategy minimizes the use of invasive treatment and toxic agents and reduces their accumulation in normal cells. Unlike traditional metal catalysts, semiconductor catalysis uses the power of excitons—electron–hole pairs generated upon light or energy absorption.^15^ These excitons play a central role in driving various redox reactions at the surface of the catalyst. Electrons and holes readily participate in redox reactions with surrounding molecules. In cancer cells, endogenously reduced substrates, such as intracellular overexpressed glutathione (GSH), can be oxidized into GSSG (≥0.24 V for GSH to GSSG) by holes, while the generated H^+^ can be converted into molecular hydrogen (H_2_) (H^+^/H_2_ = 0 V vs normal hydrogen electrode [NHE], pH = 0) by hot electrons generated on the surface of the catalyst.^16^ The generated H_2_ gas can induce cancer cell apoptosis by diffusing into mitochondria, impairing their function, and hindering adenosine triphosphate (ATP) synthesis.^17^ These agents can be converted into oxidized products by generated holes, consequently modulating the TME. Simultaneously, high generated electron levels can reduce oxygen (O_2_) and carbon dioxide (CO_2_) to produce cytotoxic superoxide (•O_2_^−^) and carbon monoxide (CO), respectively.^18,19^ However, single-component catalysts often face challenges because of the tendency of rapid exciton recombination, which significantly reduces their overall catalytic activity.^20^ Fortunately, researchers identified a promising solution: the formation of heterojunctions. These junctions, where two different semiconductors are joined, are at the forefront of developing highly efficient catalysts.^21^ Heterojunctions not only address the recombination issue but also offer an additional benefit of enhanced selectivity. The specific redox potentials of excitons can be tuned by engineering different types of heterojunctions, allowing more precise control over the desired chemical reactions. Heterojunction nanomedicine could be a promising effective cancer treatment. This approach addresses two key challenges in traditional photocatalysis: minimizing exciton recombination and enhancing selectivity. Creating heterojunctions can significantly improve the utilization of external energy, leading to more efficient generation of reactive species. Additionally, these junctions can provide multiple active sites with distinct properties, allowing better simultaneous control of targeted reactions within the TME.^22^ This targeted therapy control can potentially enhance the overall efficacy and safety of catalytic cancer therapy (Scheme 1). Moreover, metal particles commonly employed in semiconductor catalysts can serve as theranostic agents, particularly in photoacoustic (PA) imaging, computed tomography (CT) imaging, and photothermal imaging. Consequently, this review explores the basic working principles of semiconductor heterojunction catalysts. It clarifies the latest progress in designing and utilizing heterojunctions for tumor theranostics applications. Then, the review examines strategies to optimize existing heterojunctions and explores their potential for future therapeutic approaches beyond cancer treatment. It discusses the exciting prospects for integrating heterojunctions into different biomedical fields. Ultimately, this review summarizes and discusses the reported literature on exogenously activated semiconducting nano-heterojunctions in the field of cancer medicine, aiming to inspire further innovation and promote the development of heterojunctions as powerful tools for advancing biomedicine.

## II. WORKING PRINCIPLES OF SEMICONDUCTOR HETEROJUNCTION CATALYSTS

**FIG. 1.**
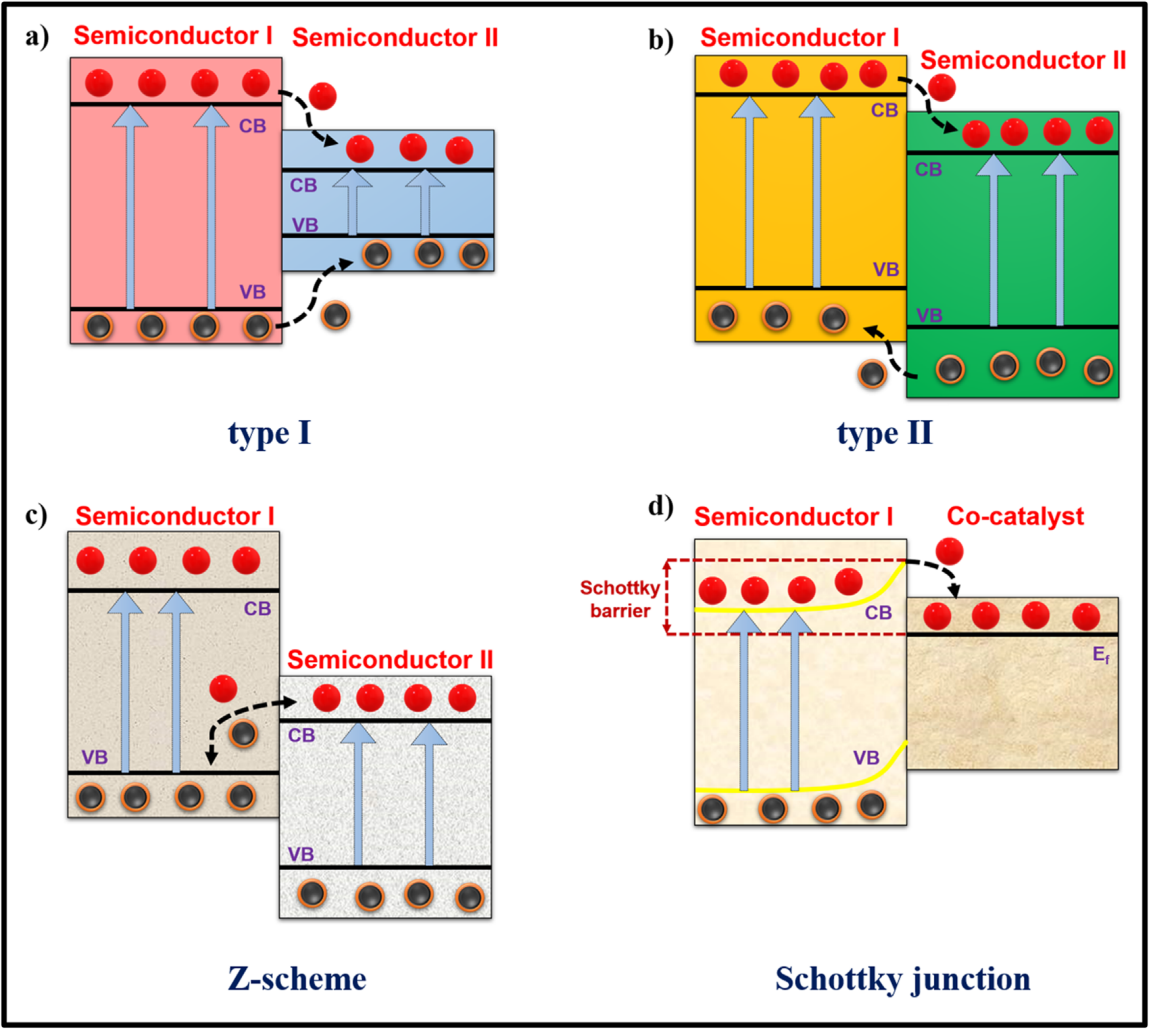
Schematic of the charge transfer mechanisms in different heterojunctions adopted in the photo/sonocatalytic process. Four types (a) type I, (b) type II, (c) Z-scheme, and (d) Schottky junction are included. CB—conduction band, VB—valence band, and E_f_—Fermi level.

Photocatalytic reactions occur on semiconductor surfaces, utilizing excited electrons and holes as catalysts for the reduction and oxidation, respectively, of target molecules. Generally, when a semiconductor receives light irradiation with energy equal to or higher than its bandgap, electrons are excited from the VB to the CB, leaving holes behind in the VB. These high-energy charge carriers on the catalyst surface can drive various redox reactions depending on the VB and CB potentials. However, in single-phase semiconductors, excited electrons are prone to recombine with holes in the VB, thereby deteriorating their catalytic power for photocatalytic applications. Several techniques in structural modification/rearrangement, including doping with impurities, heterojunction formation, defect engineering, and crystal facet engineering, were developed to address this challenge. Among them, heterojunction formation is a promising approach to enhancing photocatalytic activity.^23^ Due to band bending that occurs at the junction of two semiconductors/components, a difference in the potential develops between the two semiconductor regions. This induces a built-in electric field in the space charge zone and plays a crucial role in facilitating the spatial separation of photogenerated excitons, known as heterojunction.^24–26^ Heterojunction construction involves a combination of a semiconductor with another material, such as another semiconductor (S-S), metal (S-M), or carbonaceous material. The Fermi levels of heterostructure components align, causing band bending that hinders electron backflow and extends the lifetime of charge carriers (Fig. 1).

However, the rapid recombination of these photogenerated electron–hole pairs often limits their efficiency when using a single-phase semiconductor as a photocatalyst. Thus, researchers explored various strategies, such as element doping, compositing, and crystal facet engineering, to overcome this limitation and enhance photocatalytic activity. One of the most promising approaches involves the creation of heterojunctions by using a semiconductor with another semiconductor or a metal or a carbonaceous compound to decrease the electron–hole recombination. After hybridization, a new electronic structure is formed by the combination of materials with dissimilar band gaps. Semiconductor heterojunctions are classified into two main categories: S-S (semiconductor-semiconductor) and S-M (semiconductor-metal), which are differentiated based on the inherent electrical properties of the materials involved.

### A. Semiconductor–semiconductor heterojunctions

S-S heterojunctions are developed at the contact interface of two semiconductor materials, each with unique electronic properties and energy band structure. The unique chemical and physical properties of such materials are developed through doping or surface functionalization to form various intrinsic n- or p-type semiconductors.^27–29^ Generally, the alignment of electronic band edges and work functions of two semiconductors are the key factors regulating the charge transfer mechanism at the heterojunction interface, directly impacting the catalytic performance of materials. Based on the charge transfer mechanism, S-S heterojunctions can be classified into types I, II, and III or Z-scheme heterojunctions.^30^

In type I heterojunction with straddling band structure, the energy band alignment of two catalytic materials is completely nested within each other.^31^ As shown in Fig. 1(a), both the VB and CB of the narrow-bandgap catalyst (referred to as semiconductor II) reside within the forbidden zone of the wideband gap catalyst (referred to as semiconductor I). When exposed to sufficient energy, holes and electrons transfer from semiconductor I to semiconductor II due to a more negative CB and a more positive VB of semiconductor I.^32^ Thus, type I heterojunction, in theory, does not lead to enhanced charge carrier separation and does not improve photocatalytic activity. In a type II heterojunction catalytic system comprising two semiconductors, the CB position of one semiconductor (referred to as semiconductor I) is more negative than that of the second one [referred to as semiconductor II; Fig. 1(b)], while semiconductor II has a more positive VB position. When these two semiconductors are in contact, their Fermi levels rearrange, causing semiconductor I to donate electrons to semiconductor II. Therefore, the electrons and holes transfer in opposite directions, leading to improved charge carrier separation, reduced recombination probability, increased charge carrier lifetimes, and improved photocatalytic activity.^33^ During light irradiation, excited charge carriers are accumulated at the VB and CB of catalysts I and II, respectively. Compared to type I heterojunctions, type II heterojunctions trap excited charge carriers in two distinct spatial potential wells.^34^ This configuration improves catalytic activity by enhancing charge carrier longevity at the junction interface.

Z-scheme or type III heterojunctions exhibit a unique energy band alignment where the conduction band minimum (CBM) of one catalyst (referred to as semiconductor II) directly aligns with the valence band maximum (VBM) of the other catalyst (referred to as semiconductor I), resulting in a Z-shaped band structure [Fig. 1(c)]. This structure enables effective charge separation, enhanced redox reactions, and improved catalytic performance. In 1979, Bard introduced the concept of a liquid-phase Z-scheme heterojunction as a photocatalyst.^35^ This heterojunction catalytic system generally comprises two semiconductor components, where one acts as a catalytic center and the other serves as a redox mediator.^36^ However, liquid phase Z-scheme heterojunction catalysts face challenges, such as lower stability, limited application range due to decreased electron transfer efficiency, synthesis complexity, contamination risks, potential efficiency concerns, and material compatibility limitations. In 2001, Grätzel first adopted a direct Z-scheme mechanism to explain the photocatalytic activity enhancement in the WO_3_/TiO_2_ heterojunction. Direct Z-scheme heterojunctions are designed semiconductor interfaces that directly transfer photogenerated charge carriers (electrons and holes) between two distinct materials. This eliminates the need for an external electron mediator while maintaining the catalytic activity of both materials. It involves two semiconductors with staggered energy gaps, designated as semiconductor II (higher work function) and semiconductor I (lower work function).^37,38^ Following the contact of two semiconductors, electrons migrate from semiconductor II to semiconductor I until equilibrium in the Fermi levels is reached. Consequently, the energy bands of semiconductor II bend upward, while the bands of semiconductor I bend downward. This bending allows the excited holes of semiconductor II and the excited electrons of semiconductor I to be combined through the tunneling effect.^39^ Additionally, the internal electric field caused by Fermi-level rearrangements in semiconductors I and II minimizes the charge recombination and maximizes the utilization of excited charge carriers in redox reactions. Despite wide applicability, direct Z-scheme heterojunctions encounter challenges in selecting semiconductor materials with matched band alignment and establishing efficient charge transfer pathways at the heterojunction interface to facilitate direct Z-scheme formation over type II heterojunctions.^40–42^ Researchers address direct Z-scheme limitations by incorporating charge transfer bridges within heterostructures, enhancing efficiency, and directing interfacial charge flow.^42^ These charge transfer bridges are developed by incorporating noble metal NPs, such as Ag, Au, and Pd.^43–45^ These bridges not only modify the band alignment but also sustain the rapid charge transfer process.^46^

### B. S-M heterojunction

The oldest practical semiconductor devices use S-M heterojunctions created by combining semiconductors with metals. S-M heterojunctions are categorized in Schottky junctions [Fig. 1(d)] and Ohmic contacts and depend on semiconductor type and their relative work function. Schottky junctions are formed at the interface of metals and semiconductors, with the metal having a higher work function than the semiconductor (n-type) or the semiconductor having a higher work function than the metal (p-type). Due to the difference in the work function between metals and semiconductors, Schottky junctions effectively trap electrons and promote the separation of excited holes and electrons.^47^ Ohmic junctions occur when semiconductors with different work function values are connected to metals. In contrast to Schottky junctions, the band edges of semiconductors bend downward, allowing excited electrons to flow back to the VB and causing recombination. Hence, Ohmic junctions are not appropriate for catalysis.

## III. LIBRARY OF DIFFERENT SEMICONDUCTOR HETEROJUNCTION NANOMEDICINES USED IN CANCER TREATMENT

### A. Semiconductor heterojunction nanocatalyst for ROS-induced cancer therapy

#### 1. Photocatalysis

**FIG. 2.**
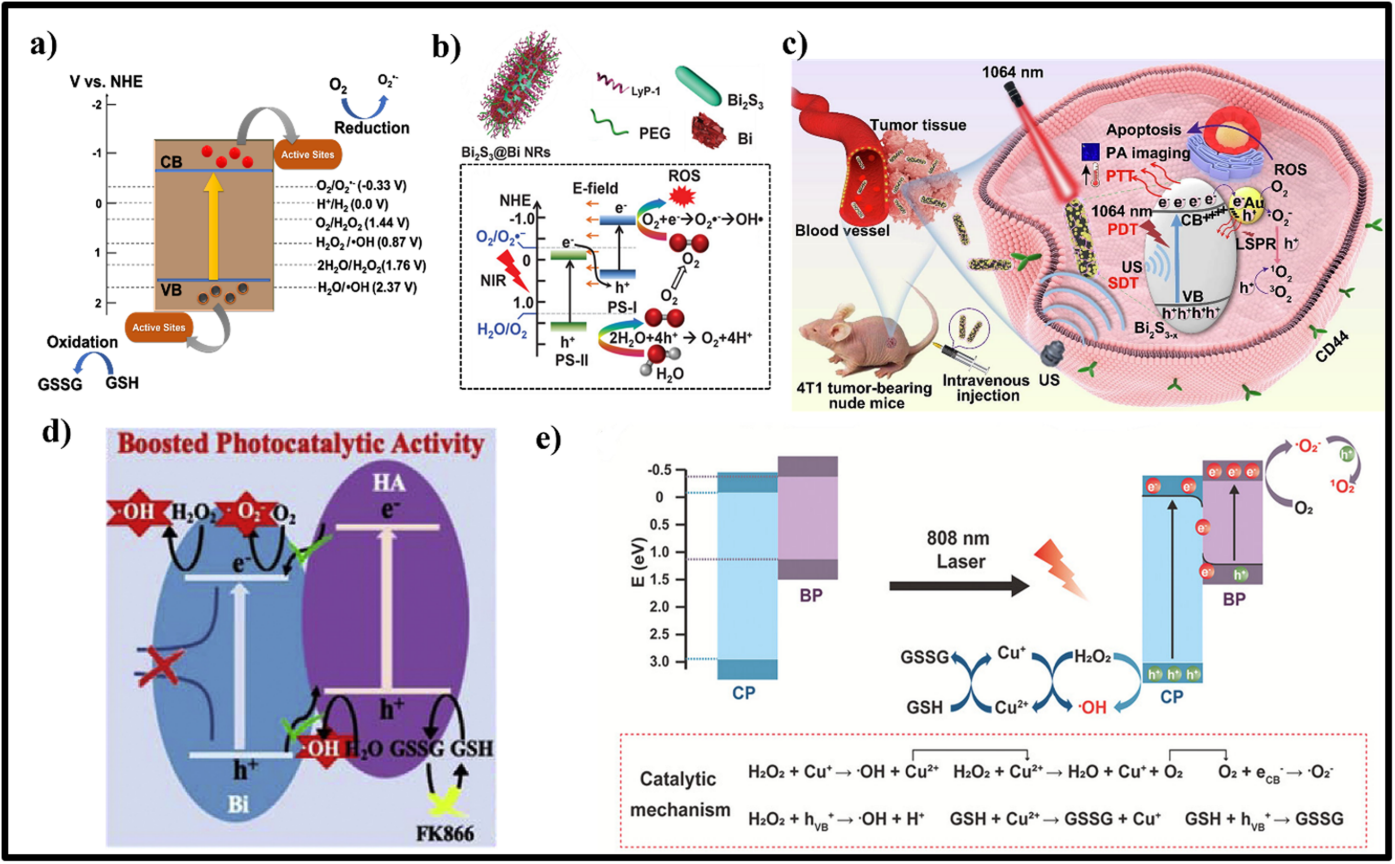
(a) Possible photocatalytic redox reactions to produce/increase ROS for oxidative damage on cancer cells. (b) Schematic representation of Z‐scheme Bi_2_S_3_@Bi heterostructure and the possible mechanism underlying the generation of O_2_ and ROS under NIR laser irradiation at 808 nm. Reproduced with the permission from Cheng *et al.*, Adv. Mater. **32**(11), 1908109 (2020). Copyright 2020 John Wiley and Sons.^57^ (c) Mechanism of Bi_2_S_3_-x-Au@HA (hyaluronic acid) for photocatalytic antitumor reactions against 4T1 tumor cells under 1064 nm NIR-II laser irradiation. Reproduced with the permission from Meng *et al.*, J. Colloid Interface Sci. **644**, 437–453 (2023). Copyright 2023 Elsevier.^58^ (d) The charge transfer mechanism and the generation of ROS in Bi-HA (humic acid)/FK866 heterojunction under NIR laser for effectively inhibiting NAD/ERK/NF-κB signal pathway and cancer cell migration. Reproduced with the permission from Song *et al.*, Biomaterials **254**, 120140 (2020). Copyright 2020 Elsevier.^59^ (e) The overall mechanisms of ROS generation and GSH depletion in Z-scheme BP@CPP heterojunctions for enhanced lipid peroxidation and amplified ferroptotic cell death. Reproduced with the permission from Liu *et al.*, Small **20**, 2309206 (2023). Copyright 2023 John Wiley and Sons.^60^

Photocatalysis is a process of utilizing light energy to initiate catalytic reactions by rearranging the electronic band structure of semiconductors. The excitation causing electron transition from VB to CB can be achieved by utilizing light energy equivalent to or greater than the bandgap energy of the semiconducting material. Thus, the generated hot electron and hole pairs facilitate semiconducting nanomaterials to engage in redox reactions at the catalyst surface. Semiconducting materials act as reducing agents when their CB potential is between +0.5 and −1.5 V (vs hydrogen electrode). VB holes demonstrate oxidizing properties when their oxidative potential is between +1.0 and +3.5 V (vs hydrogen electrode).^48^ For an effective photocatalytic reaction with minimized recombination of the charged species, the most common strategy adopted by the researchers is the inclusion of sacrificial agents. The sacrificial agents will capture the holes, enhancing the electron concentration for prolonged and efficient reduction reactions. Meanwhile, the holes oxidize the sacrificial agents and can be converted to value-added products. Researchers have employed similar strategies for treating tumors in recent years by utilizing photocatalysts and different reductive chemical factors in the tumor microenvironment. For example, Yang *et al.* used intracellularly accumulated lactic acid as a hole sacrificial agent in photocatalytic H_2_ production for NIR-induced cancer photoimmunotherapy.^49^ Meanwhile, Chen *et al.* utilized glucose as the sacrificial agent to generate H_2_ inside the diseased site.^50^ Similarly, several other intratumoral/cellular substrate molecules, such as OH^−^ (E[OH^−^/•OH] = 1.99 eV vs NHE), water (E[H_2_O/•OH] = 2.73 eV vs NHE), and H_2_O_2_ (E[H_2_O_2_/•OH] = 0.32 eV NHE), have been explored to produce •OH radicals, which is highly reactive and toxic. O_2_ molecules present inside the tumor can act as a substrate to form •O_2_^−^ by accepting the electrons from the CB of the semiconducting material (E[O_2_ /•O_2_^−^] = −0.13 V vs NHE). The generated •O_2_^−^, as the primary reactive oxygen species (ROS), can be further converted into other more potent ROS (such as H_2_O_2_ and •OH) through various approaches (•O_2_^−^+ e^−^ + 2H^+^ →2H_2_O_2_; •O_2_^−^ + H_2_O_2_ → •OH + OH^−^ + O_2_), causing substantial damage to cancer cells.^51–54^ Meanwhile, generated holes can oxidize H_2_O_2_ (E[H_2_O_2_/O_2_] = 0.69 V vs NHE) and water to O_2_ to relieve hypoxia, as well as GSH to GSSG (E[GSH/GSSG] = 0.24 V vs NHE) to facilitate the destruction of the redox TME.^55^ Therefore, photoactive nanomaterials with more negative CB (<−0.13 V) and more positive VB (>1.23 V) can produce ROS and O_2_ that are beneficial for efficient photodynamic therapy (PDT) in hypoxia [Fig. 2(a)]. Semiconducting materials with bandgap <1.53 eV can be excited with 808 nm near-infrared (NIR) laser irradiation.^22,56^ In response to the limitations imposed by rapid electron–hole recombination in single-component (unitary) semiconductors, researchers have been exploring heterojunction semiconductors as promising candidates in photocatalysis and, thus, a potential solution. Pan *et al.* put forward the initial proposal for the concept of heterojunction nanomedicine and its future.^22^ However, despite these advancements, several challenges in the exploration of heterojunction photocatalysts for tumor therapy remain.

Plants utilize solar light (photosensitization) to generate and separate charges during photosynthesis, ultimately driving catalytic reactions producing O_2_ from H_2_O. Inspired by natural photosynthesis, researchers attempted to develop artificial photosynthetic materials that can capture light, split charges, and drive valuable catalytic reactions. These artificial systems aim to mimic not only the remarkable efficiency of natural photosynthesis for O_2_ production from abundant H_2_O but also develop different materials for possible catalytic reactions, such as H_2_ and O_2_ evolution, water purification, and pollutant degradation. Inspired by solar-enabled water-splitting for clean and sustainable energy, Zheng *et al.* developed a 2D metal-free C_3_N_4_ nanomaterial as a promising water-splitting system for tumor treatment for the first time with carbon dots and protoporphyrin IX to construct a heterostructure with enhanced red-light absorption. The RGD-conjugated nanosheet heterojunction was substantially accumulated in *α*v*β*3-integrin-overexpressed 4T1 breast cancer cells. Under 630 nm light irradiation, the heterostructures accumulating within the tumor could split intratumoral H_2_O and generate sufficient O_2_ to reduce the expression of hypoxia-associated proteins, such as hypoxia-inducible factor-1*α* and carbonic anhydrase 9. O_2_ could also be partially converted to cytotoxic ^1^O_2_ with the photosensitizer, protoporphyrin IX, for effective photodynamic therapy (PDT). Overall, both PDT and hypoxia relief significantly improve the prognosis for treating hypoxic tumors.^61^ Due to the matter of concern posed by tissue penetration depth from light absorption and scattering in tissues, the delivery of visible and ultraviolet (UV) light is constrained. The penetration is dependent on light wavelength and is generally between 0.5 and 2.5 mm in healthy skin tissues making the dose availability for photosensitizer activation following tissue penetration limited.^62^ Thus, high-intensity UV and visible light irradiation cannot be employed for biomedical application because of the resulting tissue damage. Some other electromagnetic waves, such as NIR and x-rays, have good tissue penetration and are preferred for light-activated therapeutics.^63^ In Sec. II, we have discussed the structural design of various heterojunctions to enhance the separation of photogenerated electron–hole pairs. By carefully aligning the energy levels of two materials within a heterojunction, it becomes easier to direct electrons and holes into separate regions. This minimizes recombination and enhances photocatalytic efficiency, an essential factor for effective tumor treatment. Several synthesis methods have been developed to engineer these heterojunction catalysts (Table I). One widely explored method is surface oxidation, which involves oxidizing the surface of a material to form an oxide layer. This oxide layer can then interact with the core material to create a heterojunction. Surface oxidation improves adhesion between the two materials, leading to a more stable and uniform heterojunction, but also serves as a bonding interface that reduces defects and enhances the overall quality of heterojunctions. A key example is the development of surface-oxidized arsenene nanosheets (As/AsxOy NSs) with a type II heterojunction structure, used as imaging-guided, noninvasive nanomedicines for cancer therapy. Under 808 nm irradiation, photoexcited electrons in the CB of arsenene transfer to the CB of arsenic oxide (AsxOy), catalyzing the formation of ·O_2_^−^ from intratumoral O_2_. At the same time, holes in the VB of AsxOy transfer to the VB of As, facilitating the oxidation of intratumoral GSH. The heterostructure was further modified with polydopamine and a cancer cell membrane coating to enhance its biocompatibility and targeting ability. The cancer cell membrane provides a biomimetic camouflage that allows for specific recognition of cancer cells.^64^ The natural process of photosynthesis has evolved on Earth for billions of years, and chlorophyll molecules are the most abundant photosynthetic pigments indispensable for natural photosynthesis. Ou *et al.* explored a novel strategy to develop an effective photocatalysis-mediated tumor therapy using a type II heterojunction engineered with black phosphorus nanosheets and photosynthetic system of chlorophyll cells (Chls), named Chl@BP-Fe. BP nanosheets modified with polyaspartic acid (PASP) were loaded onto the surface of Chls through the chelation reaction between Fe^3+^ and PASP. When the heterojunction was exposed to NIR laser at 658 nm, the electrons in the CB of Chls were preferably transferred to the CB of BP nanosheets. Meanwhile, the holes in the VB of BP nanosheets tended to migrate to the VB of Chls. The transfer in both electrons and holes facilitated the segregation of photoexcited charges into the CB and VB of two different photosensitizers, fully preventing the recombination of electron–hole pairs. The Fe^3+^ present inside the heterostructure could both deplete GSH and catalyze the Fenton reaction to generate •OH, increasing therapeutic efficacy. Chls can also serve as an immune adjuvant by triggering T cell activation and maturation of dendritic cells, thereby facilitating immunotherapy.^65^ This study opens new avenues for researchers to explore biological and natural materials in creating novel heterojunctions.

**TABLE I.** List of different stimuli responsive semiconductor heterojunction nanocatalysts for ROS catalysis in tumor therapy.

	Materials	Synthesis method	ROS species	Band gap	Stimulus used	Modes of treatment	Year
Photo activated heterojunctions	p-MoS_2_/n-rGO–MnO_2_–PEG	Liquid exfoliation, Hydrothermal	^1^O_2_, •OH, •O_2_^−^	NA	NIR (980 nm)	PDT	2018^137^
	NIR-CD/MoS_2_	Hydrothermal	NA	1.9 eV	NIR (808 nm)	PTT	2019^138^
	CeVO_4_/Ag	Hydrothermal, Surface reduction of Ag^+^ by Ce^3+^.	•O_2_^−^	3.07 eV	NIR (808 nm)	PTT, PDT	2019^139^
	TOPY-PEG NSs	Ball grinding, Liquid exfoliation, Thermal oxidation	•O_2_^−^, •OH	FeS_2_: 0.9 eV Fe_2_O_3_: 2.1 eV	NIR (808 nm)	PTT, PDT, CDT	2019^67^
	NdVO_4_/Au	Hydrothermal, NaBH_4_ reduction	•O_2_^−^, •OH	NA	NIR (808 nm)	PTT, PDT	2019^140^
	g-C_3_N_4_-AuNP	Calcination, Photodeposition	•O_2_^−^, •OH	NA	NIR (670 nm)	PDT	2019^141^
	Bi_2_Se_3_/MoSe_2_/Bi_2_Se_3_ @PEG-Dox	Liquid exfoliation, cation-exchange	•OH	1.17 eV	NIR (808 nm)	PTT, PDT, CDT, Chemotherapy	2019^142^
	Bi–Bi_2_S_3_/BSA&FA	NaBH_4_ reduction	•OH	NA	NIR (808 nm)	PTT, CT imaging	2019^143^
	CeVO_4_/Au	Hydrothermal, NaBH_4_ reduction	•O_2_^−^, •OH	NA	NIR (808 nm)	PTT, PDT	2020^144^
	FeTiO_3_@Fe_2_O_3_	Liquid exfoliation	•O_2_^−^, •OH	NA	NIR (650 and 808 nm)	PTT, PDT	2020^145^
	g-C_3_N_4_/Cu_3_P	Calcination	•O_2_^−^	NA	NIR (980 nm)	PTT, PDT, CDT	2020^146^
	MoSe_2_/CoSe_2_@PEGs	Coprecipitation	H_2_O_2_, •O_2_^−^, •OH	1.3 eV	NIR (808 nm)	PTT, CDT	2020^147^
	NaErF_4_@ZnO	Hydrothermal, high temperature annealing	^1^O_2_	NA	NIR (980 nm)	PTT, PDT, CDT	2020^148^
	Bi_2_S_3_@Bi	Solvothermal, hydrazine treatment	•O_2_^−^, •OH	NA	NIR (808 nm)	PDT	2020^57^
	Cu_2-x_Se-Au	Oxidation-reduction	•OH	NA	NIR (808 nm)	PTT, PDT	2020^149^
	BiNS–Fe@Fe	Solvothermal	•O_2_^−^, •OH	NA	NIR (808 nm)	PTT, PDT, CDT	2021^150^
	Sb–THPP–PEG	Liquid exfoliation	^1^O_2_, •O_2_^−^	1.75 eV	NIR (808 nm)	PTT, PDT	2020^151^
	As/As_x_O_y_@PDA@M	Ball grinding, liquid exfoliation	^1^O_2_, •O_2_^−^	NA	NIR (808 nm)	PTT, PDT, CDT	2021^64^
	Bi_2_Se_3_/Au @PLGA-PEG-DOX	Hydrothermal, oxidation-reduction	^1^O_2_, •O_2_^−^, •OH	NA	NIR (808 nm)	PTT, PCT, chemotherapy	2021^152^
	M-RP/BP@ZnFe_2_O_4_	Hydrothermal, liquid exfoliation	^1^O_2_, •O_2_^−^, •OH	NA	NIR (660 nm)	PDT	2021^153^
	Mo_2_C@*N*-Carbon@PEG	Calcination	•OH	∼2.10	NIR (808 nm)	PTT, PDT,	2021^154^
	PTh@Au NCs	Oxidation-reduction	^1^O_2_, •O_2_^−^, •OH	1.80 eV	NIR (650 nm)	PDT	2021^155^
	Bi/BiOx	Regioselective coordination, orientational oxidation	^1^O_2_, •O_2_^−^, •OH	1.83 eV	NIR (660 nm)	PTT, PDT	2021^156^
	Cu_2−x_Se/Bi_2_Se_3_@PEG	Thermal injection, cation-exchange	^1^O_2_, •O_2_^−^, •OH	NA	(NIR 808 nm)	PTT. PDT, CDT	2021^157^
	CNMS	Hydrothermal, calcination	•O_2_^−^, •OH		(NIR 670 nm)	PDT	2021^158^
	MoSe_2_/Au@PEG	Hydrothermal, photodeposition	•OH	1.33 eV	NIR (808 nm)	PDT, PDT, CDT	2022^159^
	g-C_3_N_4_/rGO/ZnO-Ag	Hydrothermal, calcination	•O_2_^−^, •OH	2.6 eV	Visible-light	PDT	2022^160^
	CeO_2_@MXne	Liquid exfoliation, hydrothermal	^1^O_2_, •O_2_^−^, •OH	NA	NIR (808 nm)	PDT	2024^161^
Ultrasound activated heterojunctions	HAu-TiO_2_	Photoreduction	^1^O_2_, •OH	NA	US (1.5 MHz)	SDT	2016^162^
	Au-TiO_2_-A-TPP	Hydrothermal, NaBH_4_ reduction	^1^O_2_, •OH	2.90 eV	US (1.0 MHz and 1.5 W cm^−2^)	SDT	2019^163^
	Fe-TiO_2_	Thermal decomposition	^1^O_2_, •OH	2.3 eV	US (40 kHz, 3 W/cm^2^)	SDT, CDT	2020^80^
	Pt–TiO_2_	Hydrothermal, vacuum metal sputter deposition	^1^O_2_, •O_2_^−^, •OH	NA	1.0 MHz, 1.5 W cm^-2^	SDT, Chemotherapy	2020^96^
	TiH_1.924_	Liquid exfoliation	•O_2_^−^,^1^O_2,_ •OH	2.7 eV	40 kHz, 3 W cm^−2^	SDT PTT	2020^164^
	TiN	Liquid exfoliation	^1^O_2_, •O_2_^−^, •OH	NA	US (40 kHz, 3.0 W/cm^2^)	SDT, PTT	2021^165^
	*α*-Fe_2_O_3_@Pt	Hydrothermal, NaBH_4_ reduction	^1^O_2_	1.83 eV	US (1.0 MHZ, 1.0 W cm^−2^)	SDT	2021^85^
	Cu_2−x_O–BaTiO_3_	Hydrothermal, electrostatic interaction, calcination	^1^O_2_, •OH	NA	1.0 MHz, 1.0 W/cm^2^	CDT, SDT	2022^166^
	N-CD@LiFePO_4_	Microwave-assisted hydrothermal, ultrafast microwave reaction	^1^O_2_, •OH	NA	US irradiation (50 kHz, 3 Wcm^−2^)	SDT, CDT	2022^94^
	SMISO	Hydrothermal	^1^O_2_, •OH	NA	US (1.0 MHz, 1 W/cm^2^)	SDT	2023^167^
	Ru/TiO_2−x_@TiCN	Liquid exfoliation, hydrothermal, NaBH_4_ reduction	^1^O_2_, •OH	1.8 eV	US (50 kHz and 1 W/cm^2^)	SDT	2024^92^
x-ray activated heterojunctions	BiOI@Bi_2_S_3_@BSA	Hydrothermal	•O_2_^−^, •OH	1.63 eV	x-ray (6 Gy), and NIR (808 nm)	RT, PDT, PTT	2017^114^
	Bi_2_WO_6_	Hydrothermal	•OH	∼2.62 eV	x-ray (6 Gy)	RT	2019^111^
	Au–Bi_2_S_3_	Precipitation, Reduction	•OH	NA	x-ray (6 Gy)	RT	2019^113^
	WO_2.9_-WSe_2_	Hydrothermal, Selenization	•OH	NA	x-ray (4 Gy)	RT, PTT, CBT	2020^168^
	WO_3_@Ag_2_WO_4_@CS	Hydrothermal, deposition-precipitation	•OH	NA	x-ray (6 Gy)	RT	2020^110^
	BiOI/Bi_2_S_3_@polydopamine	Anion-exchange	•OH	NA	x-ray (6 Gy)	RCT	2021^120^
Thermoelectric heterojunctions	BST/BTS	Hydrothermal	•O_2_^−^, •OH	NA	ΔT	TET	2022^128^
	SrTiO3/Cu_2_Se	Hydrothermal	•O_2_^−^, •OH		ΔT	TET	2023^135^
	BST/CaO_2_	Hydrothermal, oxidation-reduction	•O_2_^−^, •OH	NA	ΔT	TET	2023^136^

As explained in Sec. II, in type II heterojunctions, the CB and VB of two semiconductors are staggered. This alignment in type II structure often results in reduced redox potentials because the electrons and holes are at lower energy levels after separation, which may limit the range of reactions they can effectively drive. Rather than creating type II heterojunctions, various approaches have been employed to enhance the production of ROS using semiconductor materials. In a Z-scheme heterojunction, the resulting electrons and holes possess higher redox potentials due to the electron transfer between the two semiconductors. This is crucial to driving more demanding reactions, like water splitting, where strong oxidizing and reducing agents are needed. Several approaches have been employed to construct a successful Z-scheme photocatalytic system, including those by ensuring proper band alignment, utilizing appropriate preparation methods, and achieving effective cooperation among various materials.^66^ For example, Pan *et al.* in 2019 reported the first direct Z-scheme heterojunction using thermally oxidized ultrathin pyrite nanosheets comprising FeS_2_ as cores and Fe_2_O_3_ shells. These nanosheets were synthesized through mechanical grinding and ultrasonic-assisted exfoliation. A thermal oxidation process employing a two-step probe sonication in an N-methyl-pyrrolidone (NMP)/water solution under an O_2_ atmosphere was then used to modify the surface of the FeS_2_ sheets. The sonication results in sufficient oxidization of the FeS_2_ nanosheets to form Fe_2_O_3_ shells (TOPY NSs) with a well-defined Z-scheme heterojunction. The Z-scheme electronic band structures of FeS_2_ and Fe_2_O_3_ in the TOPY NSs allow for efficient charge separation by means of the transfer of photoexcited electrons in the CB of Fe_2_O_3_ to the VB of FeS_2_, thereby preventing the recombination of electron–hole pairs in FeS_2_. The Fe_2_O_3_ shell and Fe^3+^/Fe^2+^ inside the heterostructure induced tumor tissue damage with the disruption of the redox homeostasis due to GSH oxidation and •OH production by the Fenton reaction. The generation of •O_2_^−^ from O_2_ and •OH from OH^−^ on the CB of FeS_2_ and VB of Fe_2_O_3_, respectively, was largely enhanced upon 650 nm laser irradiation. In addition, 808 nm laser irradiation generated local hyperthermia for photothermal therapy (PTT) with a conversion efficiency of 41.3%.^67^ Semiconducting heterojunction NPs are not only explored for tumor treatment but also for enhanced imaging contrast. For example, PA imaging, an emerging *in vivo* imaging technique, relies on the ability of contrasting agent to absorb light and convert it to heat. The development and optimization of these contrasting agents is obviously a vital research area with the potential to significantly enhance the capabilities and applications of PA imaging. In an S-M NP, with the heterojunction formation, photoluminescence quenching usually occurs in the semiconductor part due to increased non-radiative recombination by transferring the exciton from the semiconductor to the metallic domain. The non-radiative recombination thus generates localized heat inside the tissues, leading to thermal expansion and generation of acoustic waves, being detected as photoacoustic signals.^68^ Introducing vacancies or doping impurities and narrowing the bandgap can also enhance photon absorption and thereby the non-radiative recombination and the PA signals. TOPY NSs show excellent PA imaging due to the strong absorbance in NIR wavelength and high photothermal conversion efficiency. Moreover, the fluorescent and photothermal imaging capabilities of heteronanostructures allow them to act as promising candidates for multimodal imaging-guided cancer theranostics.^67^

Due to the large atomic nuclei and more electrons, high-Z elements, such as Au, W, and Bi, are more likely to interact with x-rays. When these high-Z elements are introduced to make a heterojunction, the resulting structures can be designed to absorb x-rays more efficiently. This is particularly useful in CT imaging, where contrast and resolution are critical. Bi_2_S_3_ nanomaterials have been recently used as PDT and PTT agents due to their higher absorption in the NIR region. The photothermal and photodynamic properties of Bi_2_S_3_ NPs can be further improved by forming heterostructures with other materials. Generally, PDT displays low efficiency in hypoxic conditions due to lower O_2_ concentration. Inspired by the VB position of Bi_2_S_3_ nanomaterials (1.48 V vs NHE), Cheng *et al.* fabricated Bi_2_S_3_@Bi Z-scheme heterostructured nanorods for photoinduced hole-mediated oxidation of water to produce O_2_ for alleviating tumor hypoxia [Fig. 2(b)].^57^ With the heterojunction formation, the electrons move from the higher Fermi level semi-metallic Bi to the lower Fermi level Bi_2_S_3_ until it reaches an equilibrium. Under 808 nm laser irradiation, the electric field stimulates the flow of electrons from the Bi_2_S_3_ to Bi, resulting in the recombination of electrons and holes in the CB of Bi_2_S_3_ and VB of Bi, respectively. This phenomenon results in the enhanced availability of holes in the VB of Bi_2_S_3_ and electrons in the CB of Bi. The free holes thus formed in the VB of Bi_2_S_3_ split water to generate O_2_. A self-supply of O_2_ and ROS production is showcased, when photoexcited electrons on the CB of Bi in Bi_2_S_3_@Bi Z-scheme heterostructured nanorods reacted with O_2_, which is deemed favorable in hypoxic tumor therapy, with an additional CT imaging capability. Generally, the conjugation of targeting ligands to the nanomaterials can enhance their accumulation in the tumor site and reduce systemic toxicity in normal tissues. PEG-SH and LyP-1 peptide with a cysteine moiety was functionalized on the surface of Bi_2_S_3_@Bi heterostructure for enhancing biocompatibility and tumor targeting. Additionally, Bi_2_S_3_@Bi Z-scheme heterostructured nanorods hold great promise for improved cancer diagnosis based on their CT imaging capability.

Various approaches have been employed to enhance the production of ROS by semiconductor nanosonosensitizers. These methods include building heterostructures, incorporating dopants, and generating surface vacancies. For example, noble metals (Au, Ag, Pd, Pt, etc.) have been deposited onto the semiconductor surface of nanosonosensitizers to create metal/semiconductor Schottky junctions. These junctions efficiently increase ROS generation by enhancing the utilization of external ultrasound mechanical energy.^69^ Vacancies are the point defects generally seen in semiconductor materials. Especially, anion vacancies are reported in metal oxides (oxygen vacancy) and metal sulfides (sulfur vacancy). These defects can alter the band structure and act as an electron trap, thereby preventing the recombination of charge carriers and altering the physiochemical properties. This leads to enhanced photocatalytic activity. Different methods have been used to engineer vacancy-rich semiconducting materials, such as introducing vacancies into pre-synthesized semiconducting material and *in situ* generation of vacancies during the synthesis procedure. Chemical reduction methods, such as material treatment with NaBH_4_, and high-temperature processes, such as annealing, have been reported to remove O_2_ atoms from the surface of photocatalysts to generate vacancies.^70^ In addition, hydrothermal, solvothermal, UV reduction, and chemical etching approaches can generate vacancies in semiconducting materials.^71^ Meng *et al.* prepared the sulfur-deficient Bi_2_S_3−x_ nanorods by one-pot hydrothermal reaction, followed by the *in situ* deposition of Au nanoparticles on the surface.^58^ The electron paramagnetic resonance signal at g = 2.004 and the binding energy reduction of sulfur in x-ray photoelectron spectroscopy spectra help them to confirm the generation of sulfur vacancy in Bi_2_S_3−x_-Au heterojunction. Bi_2_S_3−x_-Au nanorods were then surface functionalized with hyaluronic acid to target the CD44 protein selectively overexpressed 4T1 tumor. An NIR-II light-responsive sulfur-vacant Bi_2_S_3−x_-Au heterojunction was developed as an all-in-one phototheranostic platform for increased ROS generation via enhanced electron–hole separation [Fig. 2(c)].^58^ This heterojunction NPs show improved photothermal efficiency at 1064 nm laser irradiation compared to the individual components. With its magnified absorption in the NIR-II region, the PA imaging contrast is significantly improved. PDT is one of the most promising strategies for advanced combination treatment using chemotherapy. Song *et al.*^59^ reported a Bi-HA/FK866 heterojunction photosensitizer for NIR-triggered ROS generation and nicotinamide phosphoribosyl transferase inhibition. Nicotinamide phosphoribosyl transferase participates in cancer migration by activating the ERK1/2 pathway and downregulating E-cadherin. Humic acid reduces Bi (III) to elemental Bi, enabling NIR (808 nm) absorption and electron/hole pair generation due to the narrow bandgap [Fig. 2(d)]. While transmission electron microscopy confirmed its particle size (81 nm), x-ray diffraction analysis verified the formation of Bi^0^, which is responsible for elevated photothermal heating (52.7 °C, 5 min, 0.5 W/cm^2^). The Bi-HA platform facilitated efficient electron/hole separation via band alignment (Bi CB: 0.544 eV, VB: −0.986 eV; HA LUMO: −0.45 eV, HOMO: −1.284 eV). The heterojunction promoted charge separation, leading to enhanced ROS production and DNA damage in tumor cells. Photogenerated electrons reduce H_2_O_2_/H_2_O to •O_2_^−^/•OH, while holes oxidize H_2_O/GSH to •OH/GSSG. An increase in Bi-HA concentrations led to a linear increase in multi-spectral optoacoustic tomography and CT signals. Enhanced ROS generation and DNA damage increase the sensitivity to FK866 (inhibitor of nicotinamide phosphoribosyl transferase), downregulating the NAD^+^/ERK/NF-*κ*B pathway and inhibiting tumor progression. Downregulation of NAD^+^ prevents cancer proliferation and GSSG reduction through reduced NADPH expression. At the same time, reduction in ERK1/2 and NF-*κ*B expression prevents tumor migration. *In vivo* blood biochemistry showed no significant differences, indicating high biocompatibility of the Bi-HA/FK866 platform. Altogether, this study sheds light on the impact of combinatorial therapy utilizing heterojunction nanostructures, revealing its effectiveness in tumor treatment.

Beyond Bi-based heterojunctions, novel materials with heterojunction architectures have also been actively explored for photocatalytic tumor therapy.^72^ Most heterojunction nanomedicines focus on killing cancer cells by inducing apoptosis. Since anti-apoptosis is a common trait of neoplasm, the introduction of new therapeutic approaches for non-apoptotic regulated cell death is often in great demand.^73^ In a recent study, Liu *et al.*^60^ proposed PEGylated Cu^+^/Cu^2+^ doped black phosphorus (BP) and polypyrrole (CP) heterojunction (BP@CPP) NPs as a potential therapeutic approach for malignant breast cancer. TEM analysis revealed sheet-like BP@CPP (421.7 × 327.4 nm^2^) with successful Cu ion doping, confirmed by scanning transmission electron microscope and x-ray photoelectron spectroscopy analyses. BP@CPP exhibits superior light absorption across the visible light to NIR region compared to bare BP, translating to a remarkable 51.7% photothermal conversion efficiency under 808 nm laser irradiation. Its therapeutic potential lies in the engineered Z-scheme heterojunction. Upon laser irradiation, photogenerated electron and hole efficiently separate on BP and CP surfaces, respectively. This design fosters preferential recombination of photoexcited electrons in the CB of CP with h^+^ in the VB of BP. This spatial separation minimizes charge recombination, leading to high oxidative and reductive activities in the VB of CP and CB of BP, and promotes the generation of •OH and •O_2_^−^ due to favorable band potentials. Moreover, an obvious GSH depletion was achieved due to the presence of Cu^2+^ in the BP@CPP. Typically, GSH depletion results in the inactivity of glutathione peroxidase 4, leading to lipid peroxide accumulation in the cell membrane, finally accelerating ferroptosis of tumor cells [Fig. 2(d)]. Work function is an important parameter in determining heterostructure NP's band alignment. It is the minimum energy required to release an electron from the surface of a material to the vacuum (a point just outside the material). In a typical heterojunction, the difference in the work function between the two components affects the alignment of their energy bands when forming the heterojunction. This band alignment will define the charge separation and migration across the junction between two materials, which is crucial for the catalytic activity. By carefully selecting materials with appropriate work functions and electronic properties, designing an efficient nanoheterojunction is possible. A Pt/hollow-TiN Schottky heterojunction NPs was developed for 4T1 cancer therapy in a recent work.^74^ Generally, noble metals with high work function and n-type semiconductors with low work function are suitable for constructing Schottky heterojunctions. The work function and fermi level measurement by ultraviolet photoelectron spectroscopy reveal that the fermi level of hollow-TiN NPs, Pt NPs, and Pt/hollow-TiN NPs was 4.46, 4.25, and 4.34 eV, respectively, and their work function was 4.94, 5.66, and 5.13 eV, respectively. The data shows that the work function of hollow-TiN is smaller than the metal Pt, and the fermi level is higher. When these two materials form the heterojunction, the electrons will be transferred from hollow-TiN to Pt until the fermi level at the junction becomes equilibrium. At the same time, the space charge regions formed on the hollow-TiN result in the bending of the energy band upward and form a Schottky barrier. When this heterojunction was irradiated using the US, the excited electrons from the CB of hollow-TiN were transferred to the Fermi level of metallic Pt through the Schottky barrier, preventing their backflow. The holes left behind in the VB of hollow-TiN remain free without recombination. This effective charge separation enhanced the SCT performance and, thus, the ROS production and ferroptosis. Several other heterojunction NPs, such as Bi_2_MoO_6_-MXene Schottky heterojunction,^75^ l-arginine CoWO_4_/FeWO_4_ S-scheme heterojunction,^76^ and Ti_3_C_2_@ BaTiO_3_ Schottky heterojunction,^77^ have been reported to induce ferroptosis. However, photocatalytic heterojunction semiconductors face numerous challenges in their application for tumor therapy. One critical requirement is that the heterojunction materials must possess a bandgap <1.53 eV. Additionally, a significant drawback is the limited penetration of light into the inner core of the tumor, leading to a lower catalytic efficiency. Despite its challenges, phototherapy offers several advantages over other therapies, including precise cancer targeting, minimal invasiveness, and the capability of NIR-activated heterojunction catalysts to generate heat for photothermal treatment, which will be further discussed in Sec. III B. Table I summarizes additional semiconducting heterojunction materials investigated for the generation of ROS via photocatalysis for tumor therapy.

#### 2. Sonocatalysis

Sonodynamic therapy (SDT), a derivative of PDT, operates by utilizing US energy to activate sonosensitizers and O_2_ molecules for ROS production. Sonosensitizers respond to US waves by generating ROS, including •OH and ^1^O_2_. Mechanistically, ultrasound energy triggers electron transitions in sonosensitizers, causing ROS production through reactions with O_2_ and water. Important parameters that are critical for sonocatalytic therapy (SCT) are species and concentrations of therapeutically generated products and the active site of the sonosensitizer. It is vitally important to augment quantum yield and catalytic efficacy by tailoring sonosensitizer structures and properties under limited external stimulatory energy and substrate types and levels.

**FIG. 3.**
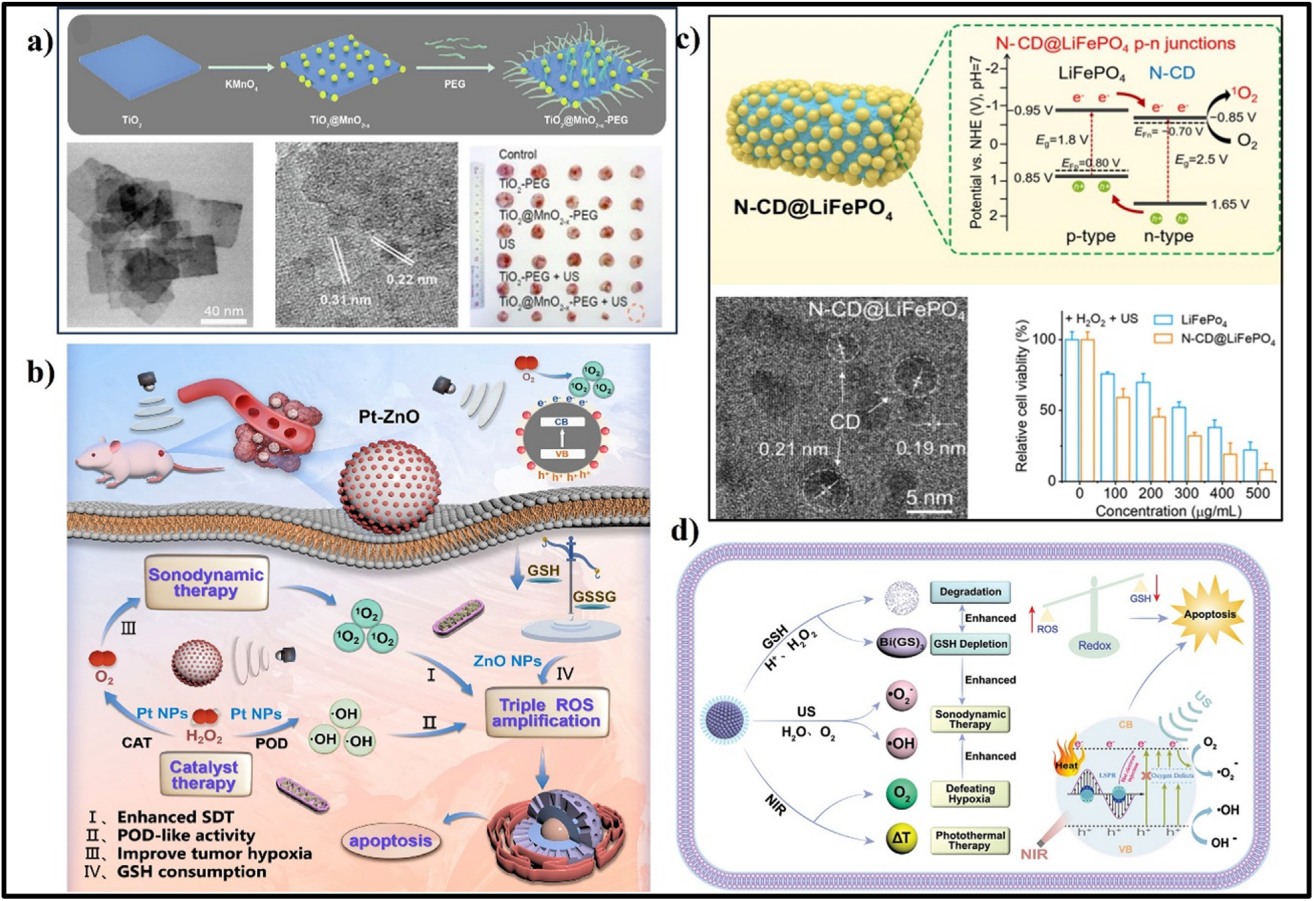
(a) Schematic illustration of synthesis procedure of TiO_2_@MnO_2-x_-PEG nanosheets, HAADF-STEM image, and HR-TEM image of TiO_2_@MnO_2-x_ nanosheets and 4T1 tumor photographs after different treatments. Reproduced with the permission from Zhou *et al.*, Chem. Eng. J. **431**, 134017 (2022). Copyright 2022 Elsevier.^81^ (b) Schematic illustration of synergistic sonocatalytic therapy of Pt-ZnO with triple ROS amplification effect for effective tumor therapy. Reproduced with the permission from Li *et al.*, Acta Biomater. **171**, 543–552 (2023). Copyright 2023 Elsevier.^88^ (c) Schematic illustration of a type II N-CD@LiFePO_4_ heterojunction and the photocatalytic ROS generation mechanism and HRTEM image featuring the deposition of CD on the surface of LiFePO_4_. Also shown is the relative cell viability of 143B cells treated with N-CD@LiFePO_4_ + H_2_O_2_ under US irradiation. Reproduced with the permission from Hu *et al.*, Chem. Eng. J. **446**, 137320 (2022). Copyright 2022 Elsevier.^94^ (d) The proposed mechanism of enhanced sonodynamic performance, including GSH depletion, hypoxia alleviation, and photothermal effect of Bi@Bi_2_O_3_@Bi_2_S_3_-PEG NPs. Reproduced with the permission from Song *et al.*, Adv. Healthcare Mater. **11**(11), 2102503 (2022). Copyright 2022 John Wiley and Sons.^95^

Inorganic sonosensitizers, such as zinc oxide, similarly produce ROS through electron/hole separation and redox reactions with O_2_ and water. This ROS-induced oxidative stress triggers tumor cell apoptosis and autophagy, contributing to tumor eradication.^78^ TiO_2_ (E_g_ = 3.2 eV) is one of the most representative materials studied in the biomedical application of inorganic sonosensitizers due to their excellent biocompatibility and high stability for *in vivo* translation.^79^ Many strategies, including heterostructure formation, have been employed to improve the therapeutic outcome of TiO_2_ NPs.^79,80^ Zhou *et al.* demonstrated an engineered TiO_2_@MnO_2_-x-PEG heterostructure that exhibited substantially improved sonocatalytic efficacy compared to pure TiO_2_ semiconductors due to enhanced electron/hole pair separation [Fig. 3(a)].^81^ Additionally, the incorporation of MnO_2_-x imparts the heterostructure with the ability to modulate tumor hypoxia, deplete GSH, and initiate Fenton-like reactions. High aspect ratio surface area and porosity are essential for better catalyst performance to absorb and oxidize/reduce the target species. In general, the porous structure enhances the light-harvesting capability of the material. It helps in the mass adsorption and diffusion of substrates and products, thus enhancing the active sites for the redox reaction and minimizing the hot electron and hole migration distance from bulk to surface.^82^ Moreover, for cancer therapy, the porous photocatalysts allow the loading of anticancer agents and deliver them to the tumor site, paving a path for combination therapy. In a study, US-activatable, metal-free TiO_2_@g-C_3_N_4_ heterostructure nanotherapeutics loaded with histone deacetylase inhibitor, romidepsin, for effective chemo/sonocatalytic combination therapy has been recently developed by He *et al.*^83^ Poly(vinylpyrrolidone) assisted solvothermal method was used to prepare hollow and porous TiO_2,_ and the total surface area of the particle was found to be 67.183 m^2^/g, with a pore diameter calculated to be 2.896 nm based on the N_2_ adsorption/desorption isotherms. Later, g-C_3_N_4_ QDs were electrostatically deposited on the surface of TiO_2_ via precipitation. The authors found that the heterojunction formation between TiO_2_ and g-C_3_N_4_ quantum dots reduced the bandgap from 3.23 to 2.98 eV, determined by extrapolating the linear part of the Tauc plot from the transformed Kubelka–Munk function. The reduction in the bandgap may promote the redox reactions under low-power US irradiation. The VB potentials of TiO_2_ and g-C_3_N_4_ QDs were 2.31 and 1.8 eV vs normal hydrogen electrode, respectively, as estimated from XPS-VB spectra. The CB potentials of TiO_2_ and g-C_3_N_4_ QDs were determined by subtracting the bandgap energy from the VB potential that gave rise to −0.92 and −1.15 eV, respectively. The band alignment led the heterostructure when it was formed to a direct Z-scheme heterojunction, allowing the migration of hot electrons from TiO_2_ to g-C_3_N_4_ QDs, yet leaving holes in the VB. The hot electrons in the CB of g-C_3_N_4_ QDs after US stimulation could effectively reduce O_2_ to •O_2−_, while the holes remaining on VB of TiO_2_ facilitated catalytic oxidation from H_2_O to •OH for effective tumor therapy. Rather than TiO_2_-based heterojunctions, several metal oxide NP-based heteronanostructures have been employed for cancer therapy.^84,85^ Metal-organic frameworks (MOF) are a type of porous coordination material built with metal ions and organic linkers and widely used in catalytic applications due to their porous architecture and efficient catalytic activity.^86^ However, the organic linkers coordinating the metal ions or clusters reduce the charge separation and thus cause rapid exciton quenching. However, this issue can be addressed by introducing metal NPs on the surface of MOF. Meng *et al.* developed Ti-based MOF (MIL) deposited with Ag for SCT.^87^ Under US stimulation, the excited electrons from the MIL move to the deposited Ag NPs by metal-to-metal electron transfer, thus effectively separating electrons and holes for effective redox reactions. Several metal nanoparticles and carbon dots exhibit POD, CAT, SOD, and GPx enzyme-like activity (nanozymes), offering considerable potential for cancer treatment. Integrating enzyme-mimicking metal NPs with semiconductors can facilitate the formation of the Schottky junction to improve the therapeutic efficacy by both semiconducting catalytic and enzymatic routes. A simple defect-rich Pt-ZnO S-M heterojunction sonosensitizer was developed by Li *et al.*, demonstrating dual nanozyme activities and excellent sonocatalytic properties, resulting in a triple ROS amplification for synergistic tumor therapy. The heterostructure formation and defects present in Pt-ZnO improved the efficiency of electron and hole separation under US, prolonging the lifespan of electron and enhancing ROS generation during SCT. Additionally, Pt-ZnO exhibited significant POD- and CAT-like activities, mitigating tumor hypoxia and increasing ROS generation [Fig. 3(b)]. Importantly, Pt-ZnO effectively depleted GSH levels, therefore minimizing ROS consumption and augmenting oxidative stress, resulting in a 614.4% increase in •OH yield and an 859.1% increase in ^1^O_2_ yield. Consequently, Pt-ZnO represented highly effective synergistic SCT, achieving a remarkable tumor inhibition rate of 98.1%.^88^ Several heterojunction NPs, such as carbon dots@Nb_2_C nanozymes (POD, CAT),^89^ Fe_3_O_4_/Ag/Bi_2_MoO_6_ NPs (POD, CAT, SOD),^90^ Co_3_O_4_@TiO_2−x_ (POD, CAT),^91^ Ru/TiO_2−__*x*_@TiCN (POD, GPx, CAT),^92^ and hollow black TiO_2_ nanosphere-carbon dots (CAT, GPx, POD like),^93^ have been reported to show the capability of acting as both nanozymes and semiconducting catalysts working together to kill tumor cells.

Recent advancements in the development of innovative sonocatalysts have opened up avenues for the creation of diverse heterojunctions aimed at enhancing the therapeutic efficacy of tumor treatment. Hu *et al.* introduced a novel biocompatible narrow-bandgap N-CD@LiFePO_4_ sonocatalyst [Fig. 3(c)].^94^ This sonosensitizer effectively catalyzed the decomposition of H_2_O_2_ into •OH radicals while simultaneously depleting overexpressed GSH through Fe^3+^ oxidation. The (PO_4_)^3^- groups significantly improve H_2_O_2_ decomposition compared to traditional iron-based nanozymes. Furthermore, the p–n junctions within the structure enhance ROS production by efficiently separating charge carriers under US irradiation. The p–n junction boosted sonodynamic therapy, Fe^2+^/Fe^3+^ mediated TME reconstruction, and targeted tumor accumulation achieved complete eradication of human osteosarcoma in a single treatment using US irradiation. Crucially, this p–n junction sonocatalyst represents a new class of semiconductor heterojunction nanomedicines that can be gradually metabolized from the body, opening avenues for their clinical translation.^94,96^ Instead of binary heterostructures, a three-component heterostructure system named ternary heterojunctions can create more complex band alignments, leading to more effective separation of charge carriers. This can reduce recombination rates and enhance the efficiency of photocatalytic reactions. In an approach, Song *et al.* developed a ternary heterojunction, Bi@Bi_2_O_3_@Bi_2_S_3_-PEG, to enhance the effectiveness of SCT, synergized by PTT, for tumors. This nano-heterojunction was prepared using the coprecipitation and reduction method. Upon US activation, the nanosonocatalyst generated abundant ROS (•O_2_^−^ and •OH), facilitated by oxygen vacancies formation, localized surface plasmon resonance effect induced by Bi, and sonoluminescence effect. These mechanisms collectively improved the electron/hole separation capability of Bi@Bi_2_O_3_@Bi_2_S_3_-PEG. Furthermore, the bismuth-based nanomaterial continuously depleted GSH, disrupting redox homeostasis and inducing sustained oxidative stress while also enabling easy degradation and metabolism. Additionally, Bi@Bi_2_O_3_@Bi_2_S_3_-PEG exhibited remarkable photothermal conversion ability upon NIR stimulation, causing thermal tumor damage and relieving tumor hypoxia while also providing endogenous O_2_ for SDT [Fig. 3(d)].^95^ Immunotherapy has advanced in the cancer treatment recently in various aspects, especially in SCT where immunogenic cell death is induced to elicit antitumor responses. Liu and his group decorated Au and carbon dots on hollow black TiO_2_, which showed a spherical hollow structure with a diameter of 100–200 nm. The heterojunction showed significant ROS generation under US due to the presence of oxygen vacancies, Ti^3+^ in the black TiO_2_, as well as the localized surface plasmon resonance of Au NPs. Hypoxia can be alleviated in TME and inhibit the immunosuppressive mediators expression by the triple-enzyme mimetic activity of the heteronanostructure to enhance the antitumor effect by SCT. Bi_2_O_3_, a wide energy gap semiconductor, has recently been employed and fabricated for combined photothermal/sonocatalytic therapy by surface decoration with Au nanorods to form a Schottky junction. Under US treatment, the hot electrons generated from Bi_2_O_3_ migrated toward metallic Au nanorods through the Schottky junction, becoming prohibited from backflow. Therefore, their recombination with holes was significantly reduced. The high electron density thereby rendered the Au nanorod of sufficient potential to reduce O_2_ to •O_2−_, while the holes in the VB of the wideband gap Bi_2_O_3_ possessed the catalytic ability to drive H_2_O oxidation to •OH. Due to the localized surface plasmon resonance (LSPR) effect of Au nanorods, the heterostructure showed good photothermal conversion under NIR irradiation at 808 nm, while Bi_2_O_3_ could deplete intratumoral GSH by forming a coordination bond. Altogether, the disruption of redox balance and photothermal heat generation by the catalytic system induces immunogenic cell death, thereby reversing tumor immunosuppressive microenvironment and promoting dendritic cell maturation and CD^8+^ T cell infiltration.^97^ Altogether, SCT using heterojunction NPs, as an emerging field in nanotechnology-assisted tumor therapy, is garnering significant attention due to its remarkable outcomes. However, further research is needed to translate the utilization of heterojunction NPs in SCT from the laboratory bench to clinical applications. Table I summarizes recent studies on heterojunction materials employed for sonocatalysis-based tumor therapy.

#### 3. Radiocatalysis

The radiocatalytic activity of nanocatalysts depends on the process and products created when materials interact physically with gamma rays or x-rays. X ray radiation can cause physical reactions in NPs, including the Auger effect, photoelectric scattering, and Compton effect, creating secondary x-rays, free electron, and fluorescence emission.^98,99^ X ray irradiation of heavy metals ejects photoelectrons with characteristic kinetic energies in the keV range. The specific energy distribution of these photoelectrons is influenced by the incident x-ray energy and the elemental composition of NPs.^100^ The generation of photoelectrons when elements interact with x-rays is directly related to their atomic number (Z) but inversely related to x-ray energy. NPs, such as Bi (Z = 83) and Au (Z = 79), can be utilized to enhance the generation of active molecules or species with therapeutic properties, thereby enhancing radiotherapy efficacy.^98,101^ When photoelectrons are emitted from lower atomic orbitals, electron from higher orbitals shift to fill the void, creating a hole and releasing excessive energy. This energy can either lead to the emission of fluorescent photons or the ionization of an electron in a higher orbital, known as Auger electron. Auger electron has low energy and limited travel distance, primarily depositing near the surface of NPs.^102^ Compton electron is generated through Compton scattering, which involves a single photon interacting with a free electron. Scintillation NPs exploit luminescence to convert x-ray energy into light. X ray irradiation excites electron (VB to CB), leading to short-lived fluorescence or long-lived phosphorescence upon relaxation.^103,104^ Various high-Z NPs, such as noble metals (Ag, Au, and Pt), rare earth elements (Gd, Tm, and Ho), and other heavy elements (Bi, Hf, W, and Ta), have been utilized as sensitizers in radiation therapy.^105–109^ Generally, x-rays interact with the above-mentioned metals (directly) and water (indirectly) to produce ROS. In recent years, inorganic semiconductor nanomaterials incorporating high-Z elements have garnered significant interest as potential radiosensitizers due to their ability to enhance therapeutic efficacy.^110^ Their mechanism of action shares similarities with photocatalysis. However, ionizing radiation excitation forms charge carriers through a more intricate pathway. Upon exposure to high-energy x-rays or gamma photons, the photoelectric effect leads to the ejection of a core electron, creating hole in the core orbital.^111^ These core holes are short-lived, rapidly relaxing to the VB within picoseconds via Auger processes. Consequently, they are not directly involved in surface reactions. The dislodged, high-energy electron (hot electron) triggers a cascade of secondary ionization and bandgap excitation events. This ultimately results in the generation of long-lived electron/hole pairs with a yield of approximately 10^2^/keV of absorbed energy in typical semiconductors. Additionally, the escape of these ionized electrons as lower-energy particles contributes to enhanced water radiolysis, further amplifying the therapeutic effect.^112^ This unique band structure enables the semiconducting nanomaterials to produce free radicals independent of O_2_ availability.^113^

**FIG. 4.**
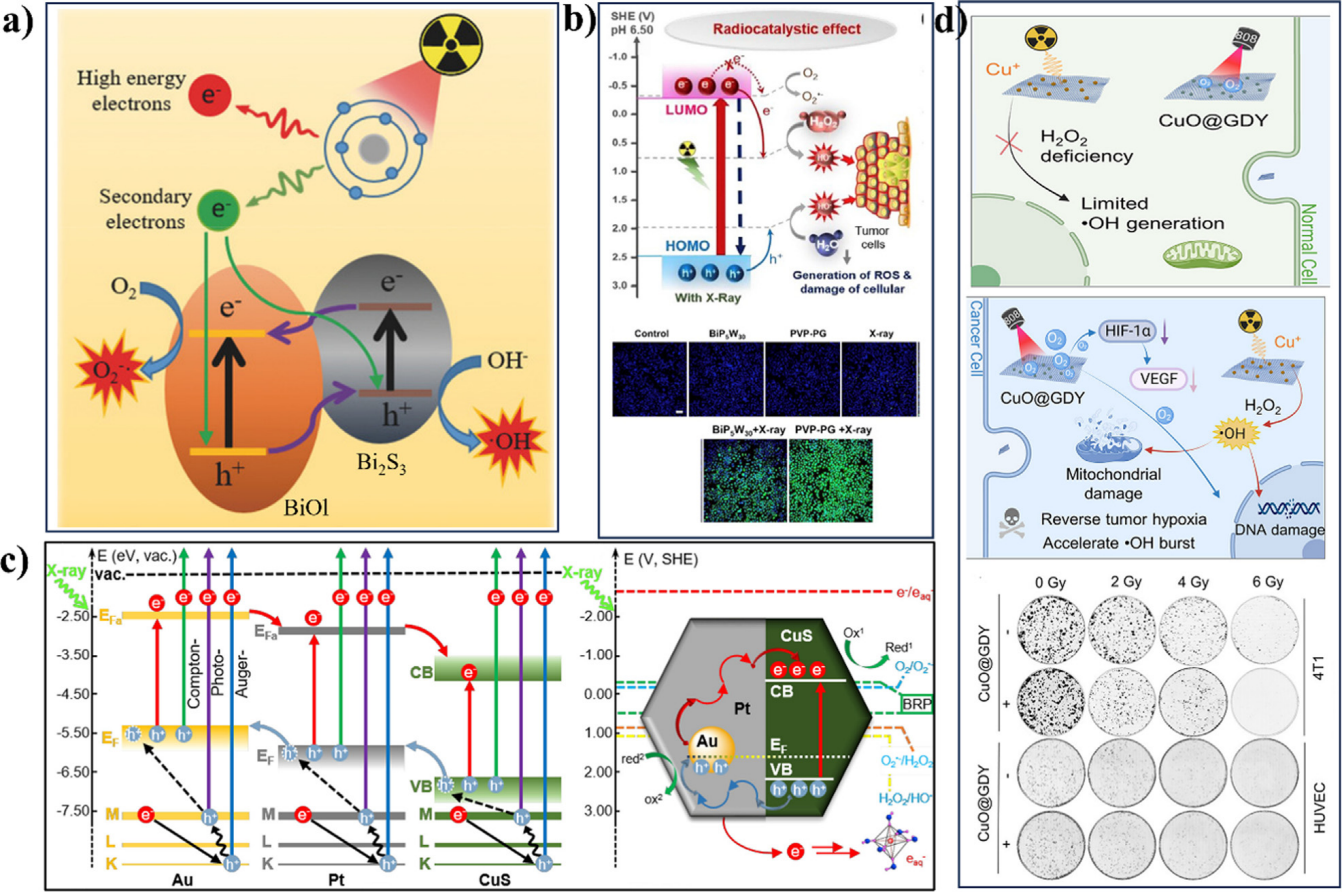
(a) Schematic representation of the generation of ROS under x-ray irradiation for radiocatalytic therapy using BiOI@Bi_2_S_3_ heterojunction NPs. Reproduced with the permission from Guo *et al.*, Adv. Mater. **29**(44), 1704136 (2017). Copyright 2017 John Wiley and Sons.^114^ (b) The possible charge transfer mechanism to generate cytotoxic free radicals in the tumor treatment by BiP_5_W_30_@rGO (PVP-PG) heterostructures under x-ray irradiation along with the *in vitro* ROS fluorescence imaging under different treatments. Reproduced with the permission from Zhou *et al.*, Biomaterials **189**, 11–22 (2019). Copyright 2019 Elsevier.^115^ (c) Illustration of possible charge transfer routes and the working principles of AuPt@CuS nanosheets to generate cytotoxic free radicals under x-ray irradiation for effective tumor therapy. Reproduced with the permission from Cai *et al.*, J. Am. Chem. Soc. **143**(39), 16113–16127 (2021). Copyright 2022 American Chemical Society.^123^ (d) Illustration of the combined radiotherapy and CuO@GDY-mediated radiocatalytic therapy and their effects on colony formation of 4T1 cells and HUVECs under various treatments. Reproduced with permission from Wang *et al.*, ACS Nano **16**(12), 21186–21198 (2022). Copyright 2022 American Chemical Society.^116^

Akin to photocatalysis being limited by material properties, employing solely single-component semiconductors might not achieve optimal results in radiocatalytic therapy (RCT). This is attributed to the intrinsic radioresistance of tumors arising from hypoxia, a characteristic feature of TME, which can induce systemic acquired radioresistance, rendering cancer cells 2–3 times more resistant to radiation compared to oxygenated (normoxic) tumor cells.^115^ Heterojunction semiconducting nanomaterials play a vital role in overcoming radioresistance and thereby enhancing treatment efficacy. Research in radiocatalysis suggests that combining metal oxide semiconductor NPs with ionizing radiation offers a potentially faster alternative to traditional photocatalysis methods using visible or UV light.^117^ Due to the emphasis on heterojunctions for enhanced radiocatalytic performance, researchers are actively investigating metal oxide/sulfide based composites with this architecture. In heterojunction catalysts, apart from the material selection, the crystal structures play a significant role in their catalytic performance. High crystallinity of the materials can contribute to better lattice matching while forming the heterojunction and promote effective charge transfer across the interface, thus enhancing the overall catalytic performance. Liquid phase deposition and chemical vapor deposition are two useful techniques employed to deposit highly crystalline and pure materials on the substrate. Although highly crystalline materials are more resistant to photo-leaching and stable under x-ray irradiation, amorphous materials may exhibit special properties in heterojunction catalysis due to their electronic characteristics. Inspired by this, amorphous/crystalline heterojunctions for effective catalytic application were built and their properties were studied.^118,119^ Generally, the amorphous phase is incorporated into the crystalline phase of the material by two strategies. One method is the amorphization of the crystalline materials, and the other is to grow the amorphous phase on the crystalline structure. One of the common routes used for the amorphization of the crystalline phase is the ion exchange method. In a recent study, Wang *et al.*^120^ developed a Z-scheme BiOI/Bi_2_S_3_@polydopamine nanosheets for RCT [Fig. 4(a)]. The nanosheets modified by glucose oxidase can generate an environment with excessive H_2_O_2_, supporting RCT. This not only aids ROS generation but also helps cut off energy to tumor cells via starvation. The energy level diagram by Wang and coworkers suggests a traditional type III exciton separation, involving significantly larger band gaps between the individual CB and VB of BiOI and Bi_2_S_3_ (0.96 and 1.68 V) compared to the gap between their combined CB and VB (0.36 V) point toward a more favorable mechanism, a Z-scheme-mediated charge transfer process. In this scenario, photogenerated electrons from BiOI CB directly recombine with hole in Bi_2_S_3_ VB. This facilitates the participation of both electron and hole in the reduction of H_2_O_2_ and oxidation of H_2_O, leading to the observed generation of •OH. *In vitro* and *in vivo* studies utilizing HeLa tumor cells demonstrated that BiOI/Bi_2_S_3_ can exploit a synergistic combination of starvation and RCT. This combined approach effectively increases ROS accumulation and chromosomal damage within tumor cell. Similarly, Guo *et al.*^114^ used BSA-coated BiOI/Bi_2_S_3_ nanoheterojunction for the combination of radio/PDT/PTT in an earlier study. BiOI/Bi_2_S_3_ offers a multimodal therapeutic approach to cancer treatment. Under x-ray irradiation, generated electron/hole pairs cause ROS production, resulting in a photodynamic effect. Additionally, the superior NIR absorption of Bi_2_S_3_ contributes to PTT. Furthermore, high x-ray attenuation and NIR absorption of the NPs make them suitable for use as a contrast agent in CT and PA imaging, enabling effective tumor visualization. *In vitro* studies using the BEL-7402 hepatocarcinoma cell line demonstrated a significant enhancement in tumor growth inhibition, highlighting the potential of this platform for cancer therapy. Recently, polyoxometalates (POMs), frequently used in the preparation of semiconducting nanocatalysts in transitional metals, such as Mo, W, and Nb, have shown wide potential in catalytic application due to their tunable composition that helps adjust the bandgap to improve the catalytic performance from catalytic active centers that are located and exposed on the surface. POMs can be easily functionalized or loaded onto several semiconducting materials for building a heterojunction nanocomposite. In yet another recent study, Zhou^115^ and coworkers have explored a novel Bi-based POM material (BiP_5_W_3_) for enhanced radiotherapy efficacy. This hybrid material combines BiP_5_W_30_ nanoclusters, containing elements with high atomic numbers, and reduced graphene oxide (rGO) to exploit synergistic functionalities. BiP_5_W_30_ nanoclusters effectively enhance the radiation-induced ROS generation in tumor cells due to their unique electronic properties [Fig. 4(b)]. Additionally, they deplete GSH, a key cellular antioxidant, via redox reactions and catalyze the conversion of H_2_O_2_ into cytotoxic •OH, significantly amplifying ROS generation upon x-ray exposure. rGO further enhances the efficacy of the platform by promoting electron/hole separation within the material, improving radiocatalysis. Moreover, the superior NIR absorption of rGO contributes to photothermal effects, elevating tumor temperature and compromising cancer cell viability. Promising results from both *in vitro* and *in vivo* studies using HeLa cells suggest the potential of this hybrid material for a combined therapeutic approach in the cancer treatment, highlighting its potential as a promising candidate for further investigation. Other Bi-based heterojunctions are also explored toward RCT, including BiVO_4_@Bi_2_S_3_ by Wang *et al.*,^121^ PVP-functionalized Bi/GO by Zhou *et al.*,^122^ and Au-Bi_2_S_3_ by Wang *et al.*^113^ These materials offer not only enhanced radiocatalytic efficiency but also proved to be useful for multimodal imaging applications owing to their high x-ray attenuation. Cai *et al.*^123^ investigated a plasmonic gold-based NPs (AuPt@CuS) for its potential in combined radiophotothermal therapy and PA/CT imaging. They highlighted the unique properties of AuPt@CuS heterojunction for enhanced therapeutic efficacy [Fig. 4(c)]. AuPt@CuS NPs demonstrated high photothermal efficiency (41.56%) due to localized electromagnetic field enhancement at the heterojunction interface, significantly rising the temperature (up to 50.1 °C) in the NIR region. This localized heating can directly ablate cancer cells. Furthermore, the favorable energy level alignment of the material facilitates the separation of radio-generated electron/hole. Then, these separated charges can interact with surrounding molecules: electron reduces O_2_ to ROS, while the hole depletes GSH, a cellular antioxidant. This depletion of GSH and the concomitant rise in ROS levels synergistically enhance the cytotoxicity of radiotherapy. Additionally, AuPt@CuS NPs exhibit high x-ray attenuation due to their composition, making them suitable for PA/CT imaging. The interaction of x-rays with NPs generates a substantial electron pool. These electrons can further react with water molecules to produce hydrated electrons that disrupt cellular redox balance, ultimately leading to tumor cell death. *In vitro* and *in vivo* studies conducted on murine mammary carcinoma (4T1) cells confirmed the therapeutic potential of this novel nanoheterojunction in cancer treatment. Photocatalysts have been widely developed to reduce the toxic metal ions in water and relieve environmental toxicity in the environmental engineering field.^124^ The same story but a different application is used to convert less toxic metal ions to higher toxic metal ions in tumor tissues for cancer treatment. For example, Wang *et al.*^116^ have constructed a Z-scheme graphdiyne heterojunction (CuO@GDY) for photocatalytic O_2_ generation and radiosensitization for tumor therapy [Fig. 4(d)]. Cu^+^, as a high-energy species, is more active to undergo the Fenton-like reaction via reaction with H_2_O_2_ than Cu^2+^. When the CuO@GDY nanocatalyst is stimulated by x-ray irradiation, Cu^2+^ ions are mostly reduced to Cu^+^ and the latter acts as an active site for accelerated Fenton-like reaction for ROS production in response to endogenous H_2_O_2_, causing tumor cell death. This combination of photo- and radiocatalytic activities leads to a controllable and precise therapy. Wang *et al.*^125^ presented a novel radiosensitizing material, a lanthanide pyrosilicate scintillator coated with titanium dioxide (LnPS@TiO_2_). This composite material leverages the unique properties of both components to achieve enhanced radiation therapy. LnPS scintillator converts incoming radiation energy into UV light. Then, this emitted UV light excites TiO_2_ NPs via fluorescence resonance energy transfer, which occurs due to the spectral overlap between the LnPS emission wavelength (368 nm) and the absorption band of TiO_2_. This efficient energy transfer significantly increases the generation of •O_2_^−^ radicals by TiO_2_. •O_2_^−^ produced by excited TiO_2_ NPs target cellular iron-sulfur clusters, disrupting essential DNA metabolic processes. Additionally, these radicals can interact with various biomolecules, such as peptides, carbohydrates, nucleic acids, and lipids, inducing long-term oxidative stress within the cell. The combined effect of impaired DNA metabolism and oxidative stress ultimately leads to tumor cell death. As discussed above, many researchers have recently described the implications of high-Z semiconductor heterojunction NPs in radiotherapy due to their versatile physicochemical properties. Table I summarizes recent studies of heterojunction materials employed for radiocatalysis-based tumor therapy.

#### 4. Thermoelectric NPs in cancer therapy

Thermoelectric phenomena bridge the fascinating interplay between heat, electricity, and charge carrier behavior within a material. Two key effects that govern this interplay are the Seebeck and Peltier effects. The Seebeck effect describes the conversion of a temperature difference into electricity, while the Peltier effect utilizes electricity to manipulate heat flow.^126^ In the nanomedicine field, there is a growing interest in thermoelectric nanomaterials utilizing the catalytic potential of electron and hole formed by temperature fluctuations near body temperature.^127,128^ For such applications, NPs with high figures of merit (ZT) are crucial. ZT is defined as ZT=S2σT/κ, where S is the Seebeck coefficient, *σ* is the electrical conductivity, T is the absolute temperature, and *κ* is the thermal conductivity. A two-pronged approach is necessary to achieve high ZT. First, semiconductor NPs with narrow bandgaps are preferred for a high-power factor (S^2^*σ*). Second, semiconductors containing heavy elements with significant lattice anharmonicity or complex structures are desirable to achieve low thermal conductivity (*κ*).^129^ For example, canonical binary compounds, such as Bi_2_Te_3_,^130^ SnSe,^131^ and PbTe,^132^ are extensively used thermoelectric materials.

Recent research in thermoelectric nanocatalysts has shown promise in cancer therapy. Wang *et al.* investigated Bi_13_S_18_I_2_, demonstrating the generation of pyrocurrent by an electrochemical workstation under the heat and cool cycle of an aqueous solution. The pyroelectric electron generated from temperature fluctuation was used to convert O_2_ to •O_2_^−^. The anticancer property was evaluated in 4T1 tumor-bearing mice, in which the synergistic effect of Bi_13_S_18_I_2_ nanorods mediated PTT and pyroelectric dynamic therapy (PEDT) selectively killed cancer cells.^127^ Building on this concept, Dong *et al.* reported the photothermoelectric property of ternary copper chalcogenide Cu_3_VS_4_ with the mitochondrial-targeting ability for PA imaging–guided synergistic photothermoelectric therapy/CDT (PTET/CDT).^133^ The development of Bi_2_Te_3_ NPs by Jiang *et al.* demonstrated the potential of photothermoelectric catalysis.^134^ Kang *et al.* engineered a p–n junction heterojunction combining SrTiO_3_ (n-type) and Cu_2_Se (p-type) to construct a new interfacial electric field to facilitate charge separation and prevent rapid recombination.^135^ Yual *et al.* utilized the exothermic reaction of CaO_2_ hydrolysis in the acidic TME to trigger the thermoelectric property of Bi_0.5_Sb_1.5_Te_3_ @ CaO_2_ NPs, release Ca^2+^ ions, and produce H_2_O_2_. The heat acts as a stimulus to excite electron to VB, and the built-in electric field minimizes charge recombination. Thus, excited electrons are used to reduce O_2_ to •O_2_^−^ radical, which in combination with Ca^2+^ ions overload increased osmotic pressure in cancer cells, ultimately leading to cell death.^136^ Ji *et al.* synthesized Z-scheme heterojunction NPs combining n-type Bi_2_Te_3−x_Se_x_ and p-type Bi_x_Sb_2−x_Te_3_ nanoplates to improve thermoelectric properties at the near-body temperature. This approach forms a new Fermi level and band bending due to the flow of electron from BST to BTS. Overall, the CB of heterojunction approached the reduction potential of O_2_ to •O_2_^−^, resulting in ROS production at temperature fluctuations at the near-body temperature.

### B. Semiconductor heterojunction nanocatalyst for photothermal therapy

Photothermal refers to a process or effect related to the conversion of light (photons) into heat (thermal energy).^169^ In the context of PTT, it specifically refers to a therapeutic approach in which light-absorbing materials (NPs) are used to convert absorbed light into heat.^170^ Then, this localized generation of heat is employed to induce hyperthermia in a targeted region, which can have various therapeutic applications, including the destruction of cancer cells or treatment of certain diseases.^171^ Recently, semiconductor nanomaterials with a wide-range bandgap have been used as photosensitizers in cancer therapy.^172^ Nanomaterials with narrow bandgap or high carrier concentration are activable under NIR irradiation, while the engineering of low carrier concentration nanomaterials or wide bandgap materials makes them a new member of NIR activable therapeutic agents in the field of cancer nanomedicine.^173^ Heterojunction formation is a popular strategy to engineer the bandgap of NPs. The formation of nanoheterojunctions induces a reduction in the bandgap of nanomaterials by modifying the electronic structure at the interface. This reduction occurs due to the alignment of energy levels between two distinct semiconductor materials, resulting in a more favorable configuration for electron transitions across the bandgap. The reduced bandgap enables the nanomaterials to absorb a broader range of photons, especially in the NIR region. The performance of semiconducting PTT agent can be improved mainly through three mechanisms: (i) increasing the absorbance and utilization of light; (ii) enhancing the electron/hole separation efficiency; and (iii) inhibiting the rapid radiative recombination of electron/hole pairs. Recent studies have shown that matched energy levels of heterostructures could significantly improve photothermal performance. Many strategies, such as doping atoms or impurities into the lattice, introducing disorder, and creating defects in traditional wide bandgap semiconductors, have been employed to modify the band energies of photocatalysts, thereby extending their activity into the NIR region.

**FIG. 5.**
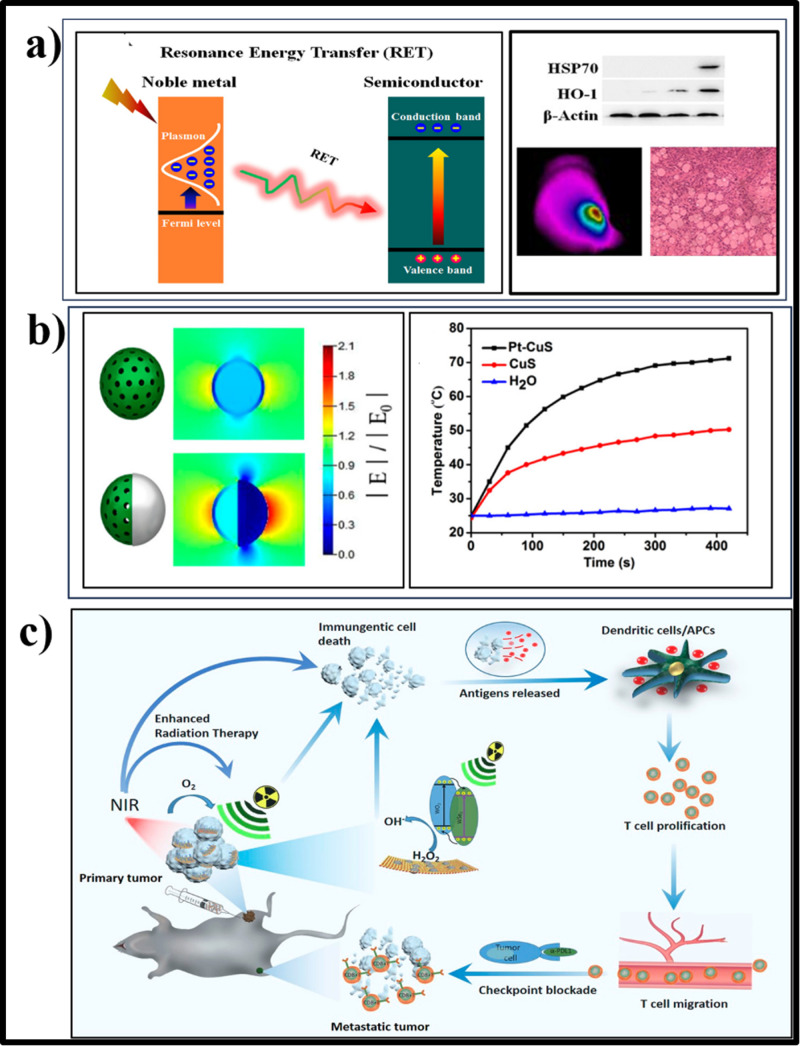
(a) Illustration of the resonance energy transfer process in Au@CuS NPs, *in vitro* western blot analysis of HSP70 and HO-1, thermal infrared images and H&E staining of Au@CuS NPs treated tumor. Reproduced with the permission from Chang *et al.*, Nano Lett. **18**(2), 886–897 (2018). Copyright 2018 American Chemical Society.^184^ (b) The FDTD simulation showing the electric field distributions of CuS and Pt-CuS under 808 nm laser irradiation for 7 min to reveal the mechanism of the photothermal effect along with experimental photothermal elevation profiles of CuS and Pt-CuS. Reproduced with the permission from Liang *et al.*, Nano Lett. **19**(6), 4134–4145 (2019). Copyright 2019 American Chemical Society.^185^ (c) Schematic illustration of photo/radiocatalytic ROS generation and immunogenic cell death for cancer immunotherapy by WO_2.9_-WSe_2_-PEG NPs under x-ray irradiation. Reproduced with the permission from Dong *et al.*, ACS Nano **14**(5), 5400–5416 (2020). Copyright 2020 American Chemical Society.^168^

The increased light absorption by the nanoheterojunction translates to a higher probability of exciting electron from the VB to the CB. The nanoheterojunction interface introduces a built-in electric field due to the difference in work function and electron affinities between the two materials. This electric field acts as a driving force spatially separating electron/hole. Then, the separated charges are available for participating in photothermal processes, such as electron transfer reactions or interactions with surrounding molecules.^174^ The efficient charge separation and reduced recombination contribute to an overall enhancement of the photothermal conversion efficiency of the nanoheterojunction.^175,176^ The absorbed energy is more effectively converted into localized heat, which is crucial for inducing photothermal ablation of cancer cells. Nuo *et al.* synthesized SnO_2_ NPs with a bandgap of 3.6 eV and doped them with Sb to obtain an NIR-responsive Sb_0.2_-SnO_2_ nanocrystal [Fig. 5(a)]. SnO_2_ originally did not respond to light in the visible or NIR spectrum due to a large bandgap; however, after Sb doping, an all-in-one semiconductor NP was formed, which was then used for NIR-II-responsive photothermal therapy in combination with CT and PA imaging.^177^ Similarly, high bandgap TiO_2_ NPs were doped with various elements, such as boron,^178^ carbon,^179^ nitrogen,^180^ iron,^181^ and tungsten,^182^ to reduce the bandgap and increase the absorption in the NIR region. Gao *et al.*^183^ synthesized TiO_2_ NPs and doped them with 15% W, increasing the free carrier concentration and leading to excellent absorbance in the NIR region. Then, WTO NPs were used to treat 4T1 by PTT and radiation therapy.

Fusing plasmonic metals or some nonmetallic semiconductors with wideband gap semiconductors is an effective approach to effective capturing of NIR light. Generally, NIR active plasmonic photocatalysts consist of a combination of plasmonic elements and semiconductors, by which the coherent oscillation of electrons within the plasmonic component can be transformed into separated electrons and holes. Under NIR irradiation, the plasmonic materials convert the incident photons into different forms of energy, including heat.^186^ Good photothermal performance of semiconductors can be generally attained by selecting materials with good light-to-heat conversion efficiency (for example, CuS NPs H_2_ was also approved as a food additive by the US Food and Drug Administration). Additionally, incorporating metal NPs with high LSPR property into the semiconducting materials is an alternative strategy to improve their photothermal property. First, the LSPR effect of metal NPs significantly enhances the hyperthermia effect under light irradiation. Additionally, the metal NPs can act as an electron sink to scavenge excited electrons from the semiconductor, preventing electrons from backflow and thus enhancing the catalytic properties. Chang *et al.* reported that resonant energy transfer in Au@CuS NPs resulted in electron/hole pair generation and improved phototherapeutic performance.^184^ Nuo *et al.*^187^ reported Nb-doped TiO_2_ with increased photothermal properties for cancer therapy. Due to the substitution of Nb^5+^ on the Ti^4+^ site, Nb-TiO_2_ NPs have high free carrier concentration in the CB, increasing the localized surface plasmon resonance (LSPR) activity of NPs due to enhanced electron transport. The free carrier concentration in 5% Nb-doped NPs was calculated using the LSPR frequency as 2 × 10^21^ cm^−3^, greater than the minimum free carrier concentration required for satisfactory absorption in the NIR region (1021 cm^−3^). Bismuth sulfide is another semiconductor NP widely used in cancer nanomedicine. However, Bi_2_S_3_ has poor LSPR due to a low carrier concentration of 1015–1016 cm^−3^. Janus nanostructures have received significant attention for combining multiple functionalities and showing the all-in-one theranostic capability [Fig. 5(b)]. Cheng *et al.* found that the crystal lattices of Bi_2_S_3_ contain S atom-deficient regions, either vacant (S vacancy) or replaced by Bi atoms (BiS antisite). The S vacancies and BiS antisite work as deep-level defects in Bi_2_S_3_ nanomaterials, promoting nonradiative recombination to emit phonons and producing photothermal properties. Following this approach, they further enhanced the photothermal conversion efficiency of Bi_2_S_3_ nanorods by depositing Au NPs on the surfaces. With the formation of the heterojunction with Au, the higher Pauling electronegativity of Au (2.54) compared to Bi (2.02) causes the lattice S atoms in Bi_2_S_3_ to bind with the Au atoms, leading to additional BiS defects and promoting photothermal effect. This work demonstrates that increasing the number of deep-level defects could increase the photothermal efficiency of semiconducting nanomaterials. Both *in vitro* and *in vivo* studies proved that the photothermal efficacy of Bi_2_S_3_ NRs could be improved by growing gold nanodots on their surface to form Bi_2_S_3_-Au NRs as a safe and practical NIR light-triggered photothermal therapeutic agent.^173^ In another study, a high-density S-vacant Bi_2_S_3−x_-Au@HA was constructed. The S vacancies reduced the bandgap (1.21 eV) of the composite and also served as an e^−^ trap. Bi_2_S_3−x_-Au@HA when compared to Bi_2_S_3−x_, the absorption band of the former extended from the NIR-I region to the NIR-II region with broad and intense absorption peaks. Bi_2_S_3−x_-Au@HA has excellent photothermal conversion efficiency reaching 43.0%, which is significantly higher than other reported agents like CuS-Au-MnO_2_ NPs (28.0%), Gd/CuS nanogel (26.7%), and CuS/MnO_2_ NPs (30.17%). Furthermore, it was even superior to photothermal agents active in NIR-I, such as CuS@Cu_2_S@Au nanohybrid (35%).^58^ A novel Bi_2_S_3_/titanium carbide (Ti_3_C_2_) two-dimensional nanoheterostructure was designed by Jiang *et al.*, who found that compared to simple Bi_2_S_3_ NPs, Bi_2_S_3_/Ti_3_C_2_-TPP significantly extended absorption to the NIR region, enhanced photocatalytic activity due to higher photogenerated carrier separation and electron transfer efficiency, and effectively accumulated in tumor cells by targeting mitochondria. These heterostructures exhibited excellent capabilities in CT imaging, rendering them highly effective as theranostic agents.^188^ A p–n heterojunction of BiOCl-Bi_2_S_3_ that promotes the effective electron/hole separation, offering a promising option to improve PTT efficiency, was used to ablate a subcutaneous hepatoma. Pt-CuS Janus heterojunction was prepared for efficient PTT. The photothermal conversion efficiencies of Pt-CuS and CuS NPs were 34.5% and 23%, respectively.^185^

Li *et al.*^189^ synthesized Pt-doped Prussian blue nanozymes for catalytic cancer therapy. To obtain high LSPR frequency, Pt (20 *μ*g/mL in terms of PB) was doped in PB, shifting the maximum absorption peak from 720 to 810 nm and improving the photothermal property by 1.7 times. PtPB showed an excellent photothermal conversion efficiency of 58.2%. Deng *et al.*^190^ prepared a heterojunction of black phosphorus and FeSe_2_, in which the lone pair of electrons on the BP surface was covalently combined with FeSe_2_ through the P–Se bond to improve the particle photostability. Heterostructure BPs-FeSe_2_-PEG showed enhanced photothermal properties due to BP with prolonged separation of photoexcited electron/hole. This heterojunction also enhanced magnetic resonance imaging contrast due to the formation of hydrogen bonds while FeSe_2_ aggregated on BP. Xu *et al.*^191^ synthesized plasmonic heterostructure of Au and metalloporphyrin NPs to prevent rapid electron/hole recombination. The high LSPR frequency of Au caused a bathochromic shift in the absorption wavelength of the heterojunction to the NIR region. Upon US and NIR irradiation, the nanoheterostructure generating ROS and hyperthermia was combined with PA imaging for theranostic cancer treatment. Dong *et al.*^168^ combined two semiconductors to form a heterojunction of WO_2.9_ and WSe_2_ NPs to obtain the WO_2.9_-WSe_2_ heterojunction that utilized x-ray and mild PTT to cure cancer, in which mild PTT induced tumor cell death and increased blood flow to the tumor for improving radiation therapy [Fig. 5(c)]. The heterojunction structure showed good photocurrent due to prolonged time in the electron/hole recombination. The heterostructure also demonstrated good photothermal efficiency and improved photostability than WO_2.9_ alone, confirming the benefit of the heterojunction. Overall, doping metal NPs into semiconductor NPs with low free carrier concentration or forming heterojunction between semiconductors to prevent electron/hole recombination improves absorption in the NIR region and enhances their photothermal efficiency.

### C. Semiconductor heterojunction nanocatalyst for gas therapy

Gas therapy is a promising cancer treatment strategy due to its high therapeutic efficacy and few side effects. However, a major challenge hindering its clinical translation is the lack of control over the distribution of gas molecules. Systemic administration often leads to nonspecific delivery, resulting in significant off-target toxicity to healthy tissues. Therefore, targeted delivery and on-demand release of therapeutic gas molecules at the tumor site are required for clinically successful gas therapy. The rapid advancement in nanotechnology made gas precision treatment possible via on-target delivery, reducing the risk of undesired toxicity. Recently, semiconductor heterojunction NPs have been employed to generate various gases in TME. The highly reductive electron (≥1.23 eV for H_2_ evolution from H^+^) generated after excitations can reduce endogenous CO_2_, H^+^, and NO_3_^−^ into CO, H_2_, and NO, respectively, offering opportunities for gas therapy.^192–195^ Meanwhile, the hole generated can oxidize H_2_O_2_ (E [H_2_O_2_/O_2_] = 0.69 V vs NHE) and water to generate O_2_, which can be employed for hypoxia relief.

#### 1. Hydrogen gas

H_2_, often considered an unreactive gas, is FDA-approved as a food additive due to its high biosafety and antioxidative properties. H_2_ gas exhibits anti-inflammatory properties by selectively eliminating cytotoxic free radicals, such as •OH and peroxynitrite (ONOO^−^), from diseased cells while keeping the functions of healthy cells unaffected. This targeted action shows its potential in treating inflammation-related diseases, including cancer, atherosclerosis, stroke, and arthritis.^196^ While the anti-inflammatory action of H_2_ eliminates •OH and peroxynitrite (ONOO^−^) in the affected cells, the physiological functions of healthy cells are unaffected.^196^ However, in tumors, the complete understanding of the apoptotic ability and cellular pathway modulation of H_2_ is yet to be clarified. Previous studies reported that the low-concentration H_2_ gas could modulate the inflammatory microenvironment. Meanwhile, its high concentrations could induce mitochondrial damage and disrupt redox homeostasis, causing cancer cell dysfunction or apoptosis.^52,197,198^ However, the lesion site, duration, and concentration of H_2_ determine the therapeutic efficiency. The practiced delivery method of H_2_ is direct inhalation, oral consumption of H_2_-rich water or capsules, and H_2_ bathing. The drawback of these approaches is their limited delivery to tumors that are challenging to access due to the high diffusion and low solubility of the gaseous molecule. H_2_ therapy combined with conventional methods to relieve harmful side effects is challenging, requiring optimization of therapeutic protocols. H_2_ nanomedicine is a potential method that introduces nontoxic nanocatalysts into lesions, thereby producing H_2_ *in situ* by triggering water-splitting reactions. Long-term H_2_ release is challenging and has limited clinical translation, underscoring the need for advanced research. In the energy sector, photocatalytic H_2_ production technology is thriving, while in practice, materials for catalyzing H_2_ production are mostly in the visible light spectrum.

**FIG. 6.**
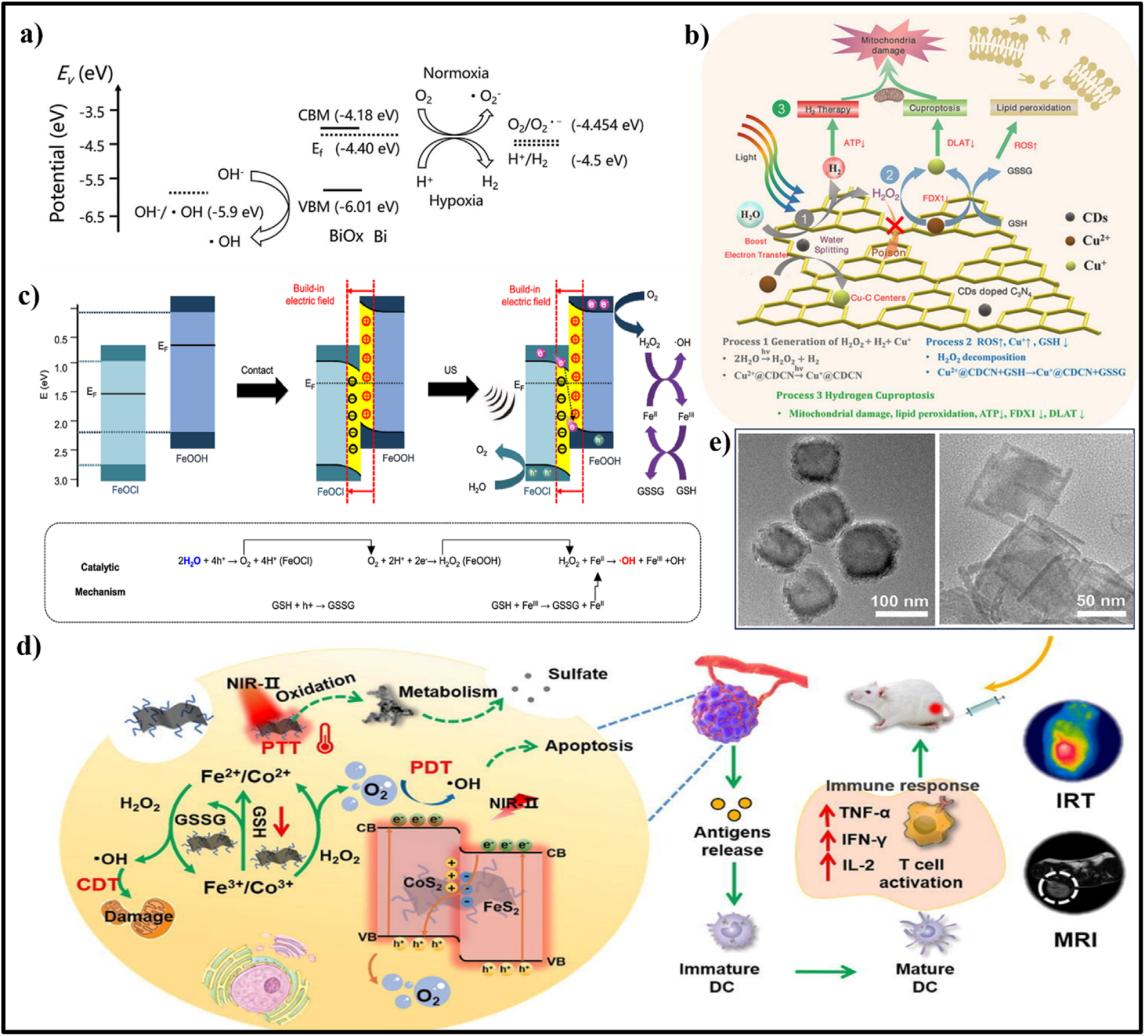
(a) Illustration of the charge transfer mechanism for catalytic ROS and H_2_ generation by Bi/BiO_x_ lateral nano-heterostructure under normoxic and hypoxic conditions. Reproduced with the permission from Qiu *et al.*, Adv. Mater. **33**(49), 2102562 (2021). Copyright 2021 John Wiley and Sons.^156^ (b) Photocatalytic mechanism of visible light driven combinatorial H_2_ therapy and cuproptosis by Cu@CDCN nanosheets. Reproduced with the permission from Ding *et al.*, Angew. Chem., Int. Ed. **62**(44), 202311549 (2023). Copyright 2023 John Wiley and Sons.^199^ (c) Sonocatalytic charge transfer mechanism in Z-scheme FeOCl/FeOOH nanosheets for O_2_ generation and H_2_O_2_ generation for effective Fenton-like reaction. Reproduced with the permission from Kang *et al.*, Nat. Commun. **13**(1), 2425 (2022). Copyright 2022 Authors, licensed under Creative Commons Attribution (CC BY) license.^201^ (d) Schematic representation of photocatalytic mechanism in FeS_2_/CoS_2_@PEG nanosheets for the production of O_2_ in combination with PTT/PDT/CDT and immunotherapy for effective tumor treatment. Reproduced with the permission from Wang *et al.*, J. Colloid Interface Sci. **625**, 145–157 (2022). Copyright 2022 Elsevier.^202^ (e) TEM image and HR TEM image of BiOBr@Bi_2_S_3_ showing the close contact of BiOBr and Bi_2_S_3_ nanorod for the effective formation of heterojunction. Reproduced with the permission from Yuan *et al.*, Adv. Sci. **11**, 2308546 (2024). Copyright 2024 John Wiley and Sons.^174^

In theory, endogenous H^+^/H_2_O can be reduced to generate H_2_ as long as CB edge of the semiconductor nanocatalyst is more negative than the redox potential of H^+^/H_2_ (vs NHE, 0 V, pH 0). GSH and lactate, which are generally overexpressed in TME can serve as a natural hole-sacrificing agents. They can deplete the holes on the surface of the catalyst consequently reducing the possibility of electron–hole recombination and enhancing catalytic H_2_ production. Zhao *et al.* developed an NIR-activatable Z-scheme SnS_1.68_-WO_2.41_ nanocatalyst that aligns with the concept of electron/hole in which catalysts generate hole as a therapeutic methodology. In a one-pot synthesis method, WO_2.41_ nanodots were heterogeneously grown over the surfaces of SnS_1.68_ nanoplates.^52^ Characterization of semiconductor heterojunction band alignment is indeed essential, as it directly influences the catalytic properties. Several techniques like x-ray photoelectron spectroscopy for evaluating the VB offset, ultraviolet photoelectron spectroscopy to assess the work function and VB maximum, photoluminescence spectroscopy for analyzing the recombination of electron–hole pairs, diffuse reflectance spectroscopy for the bandgap determination, Mott-Schottky analysis providing valuable information like flatband potential, and type of semiconductor (n-type or p-type) are some of them. The Kubelka–Munk function was incorporated into the reflectance data to obtain a plot from which the Eg of the materials was estimated. The Eg of SnS and WO in SnS_1.68_-WO_2.41_ heterojunction was found to be 1.49 and 2.43 eV, respectively.^52^ By narrowing the bandgap of SnS_1.68_, the NIR photoelectric conversion, GSH oxidation, and H_2_ production rate were enhanced. The NIR-photocurrent measurement showed that the current density of SnS_1.68_-WO_2.41_ is higher than that of SnS_2_. This suggests that under NIR irradiation, a large amount of hot electrons from WO_2.41_ are injected into conjugated SnS_1.68_ nanoplates, thereby enhancing NIR-photocatalytic reduction for H_2_ generation. This was demonstrated in a 4T1 breast cancer model where the nanocatalyst oxidized intratumoral overexpressed GSH and generated H_2_ on irradiation in a controlled and sustained manner. With the consumption of GSH and H_2_, the intratumoral redox balance was disturbed, reducing cancer cell energy and subsequently inducing DNA damage. Similarly, Pt-Bi_2_S_3_ Schottky junction NPs were developed and employed to produce a large amount of H_2_ as a gas therapy by inducing severe mitochondrial dysfunction and cell death on US irradiation–triggered activation.^16^ The authors used Mott–Schottky analysis and UV–vis diffuse reflectance spectroscopy to evaluate the band structure of the heterojunction NPs. The Eg of Bi_2_S_3_ nanorods and Pt-Bi_2_S_3_ heterojunctions were found to be 1.1 and 1.2 eV while the CB potentials for Bi_2_S_3_ and Pt-Bi_2_S_3_ NPs determined from the Mott–Schottky calculations, were −0.22 and −0.01 V, respectively. This leads to 0.89 V (1.19 V) for the VB potential of Bi_2_S_3_ (Pt-Bi_2_S_3_) NPs. This experimental analysis demonstrated that the VB of the heterojunction material is sufficient to oxidize GSH, while the CB potential showed the capability of reducing H^+^ to H_2_. Meng *et al.*^156^ utilized metallic Bi and a semiconductor BiOx to form a Schottky-type junction nanostructure for the reduction of intratumoral H^+^ to generate H_2_ with 808 nm laser, exploring the photovoltaic effect of the heterojunction [Fig. 6(a)]. Taking inspiration from H_2_ generation via solar-powered water splitting for clean energy applications, researchers developed an engineered red polymeric carbon nitride is characterized by a relatively narrow bandgap of approximately 1.71 eV alongside strong light absorption in the NIR. These characteristics facilitated efficient photocatalytic H_2_ evolution from water under NIR irradiation, resulting in the highest apparent quantum efficiency observed among the studied materials.^186^ Another group prepared Cu^2+^-anchored carbon photocatalysts by doping carbon dots (CDs) into carbon nitride (CN) in the presence of urea to produce CDCN by a thermal decomposition method. Then, they anchored it with Cu^2+^ to generate Cu@CDCN. Under visible light irradiation, photoexcited electrons split water into H_2_ and H_2_O_2_ through a two-electron reduction pathway. Interestingly, upon light irradiation, Cu^2+^ anchored on the nanocomposites was reduced to Cu^+^, activating intracellular cuproptosis, ATP and GSH depletion, severe lipid peroxidation, and aggregation of lipoylation proteins [Fig. 6(b)], thereby effectively inhibiting the tumor growth in tumor-bearing mice.^199^ The penetration of visible light to the tumor site is minimal. However, despite this limitation, NPs demonstrated notable photocatalytic efficacy both *in vivo* and *in vitro*, possibly owing to their higher efficiency. X-rays would be preferred to enhance penetration and photocatalytic efficacy in tumor tissues. Following x-ray irradiation, residual scintillation light persists temporarily, offering an opportunity for improved photocatalytic performance. Utilizing the excessive energy from x-rays and afterglow to sustain H_2_ production after irradiation cessation holds significant promise for catalytic applications within the tumor. A radiocatalytic nanotherapeutic system was developed to attain the synergistic effect of H_2_ gas therapy with radiotherapy using dumbbell-shaped Au-TiO_2_ heterostructure coated with long afterglow Co-A/Cu/ZnS NPs (Au-TiO_2_@ZnS). Under x-ray irradiation, the electrons and holes were produced in TiO_2_, and the excited electrons moved to the Au nanorod due to the p-type antiblocking layer, reducing recombination. Immediately after x-ray exposure, visible light, emitted at 578 nm from the afterglow material deposited on the Au-TiO_2_ heterostructure over 10 min, activated the Au nanorods for further hot electron generation. The hot electrons generated smoothly flowed across the metal-semiconductor Schottky barrier to the CB of TiO_2_. Separate production of hot electrons by the x-ray irradiation on TiO_2_ and exposure of the Au nanorods to visible light prolonged the H_2_ production and enhanced the efficiency of electron-catalyzed reduction reaction of protons (H^+^) in the tumor. Meanwhile, the Au nanorods improved the radiosensitivity and acted as a PA imaging agent. Altogether, the Au-TiO_2_@ZnS heterojunction structure significantly improved and prolonged the generation of H_2_ gas upon x-ray exposure for effective tumor therapy.^200^

#### 2. Oxygen gas

Hypoxia is a negative prognostic factor for solid tumors. It enhances tumor invasiveness and reduces tumor sensitivity to treatments, including PDT, radiotherapy, and chemotherapy. Supplying O_2_ is one of the most adopted techniques to alleviate tumor hypoxia. Several CAT-like nanozymes that decomposed intratumoral H_2_O_2_ to generate O_2_ have been developed and studied for tumor therapy. Recently, photo/sonocatalytic water splitting has achieved significant results in generating on-demand intratumoral O_2_ to alleviate hypoxia. Under exogenous stimulation, heterojunction catalysts can supply O_2_ through water splitting and produce ROS by reducing the generated O_2_, rendering them promising for hypoxic tumor therapy.^203^ Rather than water splitting, H_2_O_2_ has also been used as a substrate for O_2_ production in tumors by semiconducting heterojunction catalysis.^16^

Photoactive NPs can usually generate O_2_ based on their unique electronic structures. Specifically, photoactive NPs with a narrow bandgap can be excited by NIR laser to generate electron and hole in CB and VB. These holes can react with water (H_2_O) to produce O_2_ (2H_2_O + 4H^+^ → O_2_ + 4H^+^, 1.23 V vs NHE). O_2_ self-supply and ROS-producing semiconductor heterojunction NPs have been used to treat hypoxic tumors. A 2-dimensional interplanar Z-schemed FeOCl/FeOOH nanosheet was developed and adopted for intratumoral oxidation of H_2_O to generate O_2_ under US stimulation [Fig. 6(c)]. The 2-dimensional Z-scheme heterojunction with good band alignment was prepared by hydrothermal process, liquid exfoliation, and NaOH etching. The authors evaluated the effect of alkali treatment on the FeOCl/FeOOH nanosheet by spin-polarized density functional theory calculations. The fully optimized structures revealed that the Fe-Cl bond length in FeOCl is 1.72 Å, while in FeOOH, the Fe–O and O–H bond lengths are 2.03 and 0.98 Å, respectively, with a Fe–O–H bond angle of 111.5°. Substituting −Cl with −OH elongated the Fe–Cl bond from 1.72 to 1.83 Å. The authors further evaluated the interaction of water molecules with FeOCl and FeOOH. The calculated results reveal that the adsorption characteristics of water molecules with the FeOOH surface are closer than that with FeOCl, thereby indicating significant improvement in the hydrophilic properties of the material and increasing interlayer water molecule infiltration. The FeOCl/FeOOH nanosheets receiving the US stimulation induced charge separation in both FeOCl and FeOOH. The hot electrons in the CB of FeOCl recombined with the holes on the VB of FeOOH, leaving the holes in the VB of FeOCl with high oxidation potential. Meanwhile, the hot electrons left on the CB of FeOOH without recombination otherwise showed high reduction potential. This phenomenon created a built-in electric field along with the formation of a Schottky barrier at their interface, rendering the active holes of FeOCl highly effective in catalyzing H_2_O oxidation and O_2_ production.^201^ Yang *et al.*^204^ developed a novel poly(vinylpyrrolidone)-modified BiFeO_3_/Bi_2_WO_6_ with a p–n type heterojunction for the reconstruction of the immunosuppressive TME. Irradiation with 660 nm light generates holes that trigger intratumoral H_2_O_2_ decomposition, boosting intratumoral O_2_ levels and alleviating tumor hypoxia. This multi-pronged effect enhances PDT and radiotherapy, while promoting a switch in tumor-associated macrophages from immunosuppressive M2 to immunostimulatory M1 phenotype. Additionally, BFO/BWO-PVP nanoparticles demonstrate excellent performance as a CT imaging contrast agent. However, the depth penetration of 660 nm into the tumor remained an obstacle to achieving maximum efficacy. In another work, An NIR-II-responsive FeS_2_/CoS_2_@PEG (FCs@PEG) Z-scheme heterostructure was developed for intracellular O_2_ generation to alleviate hypoxia in cancer treatment [Fig. 6(d)]. The formation of the heterostructure by FeS_2_ and CoS_2_ established a Z-scheme heterojunction through band bending, resulting in the migration of electrons from the CB of FeS_2_ to the VB of CoS_2_ under NIR-II activation. The holes left in the VB of FeS_2_ exhibited an oxidation potential of 1.31 eV, sufficing to oxidize H_2_O to O_2_.^202^ Yuan *et al.*^16^ designed Bi_2_S_3_@Pt Schottky heterostructure for sonocatalytic tumor therapy. Pt NPs decorated over Bi_2_S_3_ nanorods catalyzed the excessive H_2_O_2_ in the TME, generating O_2_. Furthermore, the sonoexcited holes could also oxidize H_2_O_2_ to generate O_2_, alleviating tumor hypoxia. In addition to transforming tumor-associated M2 macrophages into M1 phenotype, the production of O_2_ alleviated the hypoxic TME and downregulated the expression of HIF-1*α* to inhibit tumor angiogenesis, demonstrating a successful reversal of immunosuppressive TME. Overall, O_2_ is produced by heterojunction catalysts via H_2_O or H_2_O_2_ oxidation in the TME by reactive electron/hole.

#### 3. Carbon monoxide

Carbon monoxide (CO) is an endogenous signaling molecule studied in preclinical studies using multiple experimental models. CO is a promising therapeutic agent that benefits the treatment of diseases, including cancer, by increasing its endogenous production and exogenous delivery.^205^ Since CO has a high affinity toward hemoglobin but no tumor selectivity, direct administration poses a threat. Therefore, sophisticated techniques should be implemented for *in situ* CO generation in tumor tissues. Many approaches are under investigation, among which photocatalytic CO_2_ reduction has emerged as a promising method. CO produced by photocatalysis sensitizes cancer cells to chemotherapy while shielding healthy cells. In a recent study, Ag_3_PO_4_-doped CD-decorated C_3_N_4_ NPs and functionalized with histidine-rich peptide (HisAgCCNs) were prepared as a Z-scheme system to convert CO_2_ to CO under 630 nm light irradiation. The CO production rate of HisAgCCNs reached 65 *μ*mol/h^−1^/g mat^−1^, significantly increasing the cytotoxicity of anticancer drug DOX by 70%.^206^ However, the limited penetration of the light, low internal CO_2_ concentration, and the strong dependence on internal CO_2_ hinder efficient CO_2_ photoreduction *in vivo*. Thus, developing novel photocatalytic CO generation systems to overcome these problems is essential. Wang *et al.* developed a bicarbonate-conjugated nanocatalytic system to generate CO gas for tumor therapy. They prepared defective WO_3_ nanosheets (DW) decorated with lipoic acid–conjugated dopamine (LA-DPA) through W-S bonds. Then, they conjugated ferric ions as a coordination center to bridge both DPA and bicarbonate and termed it P@DW/BC. Upon 808-nm NIR laser irradiation, DW nanosheets serve as a PTT agent and a catalyst to convert CO_2_ to CO. The CO-mediated anti-inflammatory effect greatly improved the survival rate of mice after PTT.^207^

US-assisted catalysis has recently become a mainstream treatment strategy primarily due to its operability and penetration depth. Recently, BiOCl/Bi_2_O_3_ was developed by wet chemical method by integrating interplanar heterojunction synthesis and 2D ultrathin heterojunction exfoliation in single step. The active catalytic sites located at the CB (−1.1 eV) of BiOCl could catalyze the generation of •O_2_^−^ (O_2_ + e^−^ → •O_2_^−^) and CO (CO_2_ + 2H^+^ + 2e^−^ → CO + H_2_O). Meanwhile, the VB (0.4 eV) of Bi_2_O_3_ could catalyze the generation of •OH (H_2_O + h^+^ → •OH + H^+^). Based on US excitation, BiOCl NPs and BiOCl/Bi_2_O_3_ nanosheets catalyze CO_2_ reduction, and BiOCl/Bi_2_O_3_ nanosheets exhibited more efficient CO generation than BiOCl NPs. Enhanced CO yields were observed in hypoxia.^208^ The CB values of BiOCl and Bi_2_O_3_ were −1.1 and 0.4 eV, respectively, and the E_0_ reduction of CO_2_/CO (−0.53 eV) was lower than that of the CB of BiOCl. In addition to material properties such as crystallinity, porosity, surface area, and surface energy the orientation and interfacial connection of the constituent materials within a heterojunction should also be considered as one of the critical factors.^209,210^ Xiong *et al.* fabricated three different heterojunction interfaces with n-type *α*-Fe_2_O_3_ and p-type Bi_2_O_3_.^210^ The three heterojunctions fabricated were ring-to-face (I), face-to-face (II), and rod-to-face (III), respectively. The photocatalytic ability of the heterojunctions was superior to *α*-Fe_2_O_3_, and the photocatalytic ability among the heterojunctions was on the order of face-to-face > rod-to-face > ring-to-face. The authors found out the reason behind the superior catalytic property as the interfacial connection of face-to-face was superior to that of rod-to-face and ring-to-face by electrochemical impedance spectroscopy. This study has highlighted that constructing an efficient heterojunction for photocatalysis requires matching the band positions and considering the interfacial connection between the materials. In the work of Yuan *et al.*,^174^ a BiOBr@Bi_2_S_3_ S-scheme heterojunction was developed for US-triggered CO therapy. Bi_2_S_3_ nanorods were grown on the edges of BiOBr nanosheets for better interfacial connection and superior catalytic ability [Fig. 6(e)]. BiOBr nanosheets were prepared by hot injection method and the length of the Bi_2_S_3_ nanorods were adjusted by altering the concentration of thioacetamide. Similar to type II heterojunctions, the S-scheme heterojunction also consists of both reduction and oxidation catalysts but entirely differs in the charge transfer route from the type II counterparts. In the S-scheme heterojunction, the reduction catalyst has higher CB and VB, as well as a smaller work function, compared to the oxidation catalyst. When these two semiconductors are in close contact, electrons in the reduction catalyst spontaneously diffuse to the oxidation catalyst, creating a significant electron gradient between the electron depletion layer and electron accumulation layer near the interface in the reduction catalyst and oxidation catalyst, respectively. This gradient accounts for the Fermi level bending in the interface region [Fig. 6(e)].^174^ In the BiOBr@Bi_2_S_3_ S-scheme heterojunction, the CB of Bi_2_S_3_ (−0.49 V) acted as the reduction site for electron-catalyzed conversion of intracellular CO_2_ to CO for CO therapy, while the VB of BiOBr (2.5 V) served as the oxidation site to convert water into cytotoxic •OH radicals. Thus, the attained CO gas effectively damaged mitochondria and reduced intracellular energy production, synergistically inducing tumor cell apoptosis in cooperation with cytotoxic •OH radicals.

## IV. CONCLUSIONS AND FUTURE PERSPECTIVES

Despite considerable progress in patient care, the global incidence of various cancer types continues to grow. Developing safer and more efficient anti-cancer treatment approaches are of great demand. In recent decades, nanotechnology has emerged as a promising and innovative medical approach for cancer diagnosis and treatment. In the hunt for cancer, nanomedicine has been diversified into different fields. Among them, heterojunction tumor nanomedicine is a burgeoning field that involves multiple disciplines. Herein, we summarize and explain the recent developments and achievements in exogenous-activated nano-heterojunction for tumor treatment. The first part of this review article includes the fundamental aspects and working principles of different heterojunctions. The latter consists of the summarized report on applying literature in heterojunction semiconductor nanocatalysts as cancer theranostics with specific examples and detailed mechanisms. In short, Exogeneous stimuli activated heterojunction catalytic therapy activates *in situ* tumor-localized catalytic reactions through nontoxic or low-toxicity nanocatalysts in response to external stimuli (light, US, or temperature), converting substrates in the TME into effective therapeutic agents to induce tumor cell death without affecting normal tissues. This emerging technology offers promising avenues for alternative therapeutic modalities, such as PTT, radiotherapy, gas therapy, and starvation therapy, to cancer treatment. However, several factors control the efficacy of heterojunctions. For the effective charge separation and an inbuilt electric field formation at the interface, a direct contact between the components is essential. For effective redox reactions inside the TME, The CB and VB positions plays a crucial role in the effective transfer of electron/hole and are closely associated with the type of heterojunction achieved. The built-in electric field and potential barriers at the heterojunction interface not only rely on the band positions but also other factors, such as semiconductivity (n- or p-type), work function and Fermi level. The advancement of research in this field is implementing heterojunction nanoparticles as a promising new candidate for cancer treatment. However, the clinical translation of heterojunction catalyst is in its infancy and requires further substantial progress since severe concerns exist.

Below is a list summarizing the factors that need to be addressed to maximize the effectiveness of heterojunction-based catalytic systems from laboratory scale to clinical application.

### A. Rational alignment of energy bands

Improper band alignment can create barriers to electron and/or hole transfer, leading to the recombination of charge carriers at the interface and reducing the photocatalytic efficiency. Achieving the desired band alignment often requires careful selection of materials and precise control of their interface. This alignment ensures that the energy levels of the materials involved are appropriately matched, enabling efficient charge transfer. When the energy bands of different materials in the heterojunction are favorably aligned, it facilitates the separation of photogenerated electron/hole pairs, which is crucial for the catalytic activity. Second, efficient migration of these photogenerated electron/hole pairs is vital for catalytic performance. Effective transport of charge carriers within the heterojunction ensures they reach the catalytic sites promptly, minimizing recombination losses and maximizing the utilization of photogenerated charges for catalysis. The orientation and interfacial contact between the two components forming the heterojunction should be considered along with the band matching to enhance catalytic performance. The prior criteria for constructing a desirable heterojunction include careful selection of materials and preparation methods with appropriate band structure and bandgap matching. Utilization of theoretical models (for example, density functional theory calculation) and simulation of heterojunctions to predict and optimize the band alignment before experimental implementation would be helpful in the construction of an effective heterojunction.

### B. Integration of different materials with different crystal structures

One of the main challenges in fabricating heterojunctions is the integration of materials of different crystal structures as lattice mismatches and defects at the interfaces often occur. These defects can act as recombination centers for charge carriers, reducing the efficiency of the heterojunction. To address this issue, it requires precise control over the synthesis conditions, including temperature, pressure, and techniques such as atomic layer deposition or molecular beam epitaxy, which allow for the growth of high-quality heterojunction with minimal defects. The surface properties, porosity, and crystallinity of heterojunction materials also play crucial roles in determining their effectiveness. Surface properties, including surface charge and functional groups, influence the interactions with biological environments and the stability of the NPs. Porosity affects the diffusion of reactants and products and the availability of active sites for catalysis. Crystallinity, on the other hand, impacts the charge mobility and the ability of the heterojunction to maintain efficient charge separation. High crystallinity typically enhances charge transport, while poor crystallinity often induces recombination losses. However, recent research has shown that using amorphous materials fused with crystalline materials exhibited superior efficacy. While the design and fabrication of heterojunction nanoparticles for cancer therapy present several challenges, particularly in integrating different crystal structures, advancements in fabrication techniques and a better understanding of the material properties can lead to successful development of highly effective therapeutic agents. The interactions between catalysts and reactants predominantly occurs on the surface or at interfaces, rendering material efficiency partially reliant on specific surface area. A larger surface area provides more active sites for reacting with absorbed water and other biomolecules to execute redox reactions. A complex interplay of physical and chemical properties influences the overall efficiency of heterojunction photocatalytic systems. While it is well-established that these factors impact photocatalytic performance in various ways, comprehensive and detailed analyses that encompass all these aspects are still lacking.

### C. Size, shape, and stability of the heterojunction materials

Additionally, ensuring the size, shape, and uniformity of heterojunctions is paramount for consistent and reliable catalytic performance, intratumoral accumulation, and anticancer activity. Irregularities or variations in the shape and size of heterojunction components can cause differences in catalytic activity across the material, resulting in reduced overall efficiency. Meanwhile, the NP size is a critical factor in determining the blood circulation time and bioelimination, the important parameters strongly associated with the material therapeutic efficacy. NP size ranging from 20 to 200 nm is widely accepted since these nanoparticles can passively extravasate through the leaky tumor vasculature and preferentially accumulate in tumors due to the enhanced permeability and retention effects. The stability of heterojunction is crucial for the application in cancer therapy. Issues concerning aggregation or interactions with biological components could impair the heterojunction's effectiveness in targeted applications, such as tumor treatment. Surface functionalization is a viable solution to enhance the stability of heterojunctions during systemic circulation. Modifying the surface of the heterojunctions with biocompatible polymers or targeting ligands and proteins could improve its resistance to degradation, prevent aggregation, and facilitate specific interactions with target cells or tissues. The interface between the heterojunction materials should remain intact and effective during the treatment period inside the TME. Any degradation at the interface, particularly under acidic tumor conditions, can significantly compromise the overall performance of the heterojunction in tumor treatment applications, which should be considered while designing heterojunctions.

### D. Selection of exogenous stimuli

The limited ability of external stimuli, such as light, to penetrate biological tissues often necessitates a significant amount of nanocatalysts for optimal efficacy. However, this approach can introduce various biosafety concerns. Consequently, there is a growing interest in combining different therapies, such as PDT and SDT, or radiotherapy, to utilize their respective advantages. The development of multifunctional sensitizing nanomaterials for sonophototherapy holds considerable promise. By integrating PDT and SDT into a collaborative treatment approach, sonophototherapy not only addresses the issue of low tissue penetration but also achieves therapeutic efficacy with a reduced dose of nanomaterials. This combined therapy is expected to be a significant research focus in the future, offering enhanced therapeutic outcomes while mitigating safety concerns. X-rays have significant tissue penetration capability, allowing them to reach deep-seated tumors. They can penetrate the tumor to activate the catalytic nanoparticles, making radiocatalysis an effective strategy against deeply set tumors. However, high doses of x-rays can cause collateral damage to adjacent healthy tissues during the cancer treatment. Consequently, developing nanoheterojunctions with high catalytic efficiency that necessitate low dose x-ray may offer a promising solution.

### E. Biosafety and biocompatibility

Nanoheterojunction materials, often entailing metal-based semiconductors, are constrained to laboratory research alone due to toxicity and biosafety issues. One of the major requirements for the clinical translation of heterojunction catalysts is to ensure biocompatibility in particular by means of minimizing off-target effects. To reduce off-target, various surface modifications with biorecognizable ligands have been employed to impart active targeting. Despite acquiring target specificity, selectivity and sensitivity must be enhanced for preferred biodistribution. For specific control over the location and timing of activating therapeutic agents, exogenous stimulation helps in the controlled activation, thereby minimizing off-target toxicity. In addition, more studies should be carried out on low-dose heterojunction materials with high elimination rate from the body. Currently, the biosafety study centers on the short-term toxic effects in the laboratories. However, a substantial and comprehensive study of cytotoxicity, genotoxicity, and long-term effects is required to take this promising approach further. The unpredictable variation in the level of substrates undergoing catalytic redox reactions as the onset of the treatment remains a great challenge in tumor-specific and patient-specific therapy. Due to their wideband gap, certain heterojunction materials generate high-energy electrons and holes. A major concern ensues with their broad and nonspecific reactivity with proteins and biological molecules. Extensive research and new material design on the specificity of heterojunction catalysts are important to divulge the potential of heterojunction in biomedicine. Other challenges are the high cost and complexity of manufacturing heterojunctions. The fabrication process often requires specialized equipment and techniques, which can be difficult to scale up for mass production.

Therefore, multidimensional modulation of heterojunction nanocatalyst structures and properties to maximize quantum yields and catalytic efficiencies under limited external stimulative energy and substrate types and concentrations is key to elevating the clinical potential of nanocatalytic therapy. By addressing these requirements, researchers can enhance the catalytic efficacy of heterojunction-based systems, unlocking their full potential for various biomedical applications.

## Data Availability

Data sharing is not applicable to this article as no new data were created or analyzed in this study.
